# Characterization, Bioactivity, and Biodistribution of 35 kDa Hyaluronan Fragment

**DOI:** 10.3390/life14010097

**Published:** 2024-01-08

**Authors:** Munkh-Amgalan Gantumur, Xiaoxiao Jia, Jessica H. Hui, Christy Barber, Li Wan, Lars R. Furenlid, Diego R. Martin, Mizhou Hui, Zhonglin Liu

**Affiliations:** 1 College of Life Sciences, Northeast Agricultural University, Harbin 150030, China; mnkh.calm@gmail.com (M.-A.G.); 15621486915@163.com (X.J.); jhhui@alumni.stanford.edu (J.H.H.); 2 Department of Medical Imaging, The University of Arizona, Tucson, AZ 85724, USA; cbarber@arizona.edu (C.B.); liwan63@gmail.com (L.W.); furen@radiology.arizona.edu (L.R.F.); 3 Houston Methodist Research Institute, Houston Methodist Hospital, Houston, TX 77030, USA; drmartin@houstonmethodist.org

**Keywords:** hyaluronan (HA), low-molecular-weight HA (LMW-HA) fragment, cell migration, inflammatory biomarkers, immune cells

## Abstract

It has been reported that hyaluronic acid (HA) with a 35 kDa molecular weight (HA35) acts biologically to protect tissue from injury, but its biological properties are not yet fully characterized. This study aimed to evaluate the cellular effects and biodistribution of HA35 compared to HA with a 1600 kDa molecular weight (HA1600). We assessed the effects of HA35 and HA1600 on cell migration, NO and ROS generation, and gene expression in cultured macrophages, microglia, and lymphocytes. HA35 was separately radiolabeled with ^99m^Tc and ^125^I and administered to C57BL/6J mice for in vivo biodistribution imaging. In vitro studies indicated that HA35 and HA1600 similarly enhanced cell migration through HA receptor binding mechanisms, reduced the generation of NO and ROS, and upregulated gene expression profiles related to cell signaling pathways in immune cells. HA35 showed a more pronounced effect in regulating a broader range of genes in macrophages and microglia than HA1600. Upon intradermal or intravenous administration, radiolabeled HA35 rapidly accumulated in the liver, spleen, and lymph nodes. In conclusion, HA35 not only exhibits effects on cellular bioactivity comparable to those of HA1600 but also exerts biological effects on a broader range of immune cell gene expression. The findings herein offer valuable insights for further research into the therapeutic potential of HA35 in inflammation-mediated tissue injury.

## 1. Introduction

Hyaluronic acid (HA), also called hyaluronan, is a naturally occurring, anionic, and nonsulfated glycosaminoglycan widely distributed throughout human tissues [1]. As a significant component of the extracellular matrix (ECM), HA reinforces a complex network of biomolecules that support tissues and organs. HA exhibits various molecular weights due to its varying polymer size. High-molecular-weight HA (HMW-HA), typically defined as having a molecular weight (MW) greater than 500 kDa, is used extensively in the medical field, such as in the treatment of knee osteoarthritis through local injections into the joint cavity [2,3,4,5]. HMW-HA is characterized by strong viscoelasticity, low water solubility, and poor permeability in human tissues; it neither dissolves easily in water, nor passes quickly through human tissue [6,7]. Gel-like HMW-HA has been shown to improve the health span of mice by ameliorating age-related inflammation [8]. In contrast, low-molecular-weight HA (LMW-HA) (MW < 500 kDa) demonstrates higher tissue permeation [2,3,4,5]. As previously reported, a permeation assay conducted over 15 min revealed that the amount of LMW-HA permeated at 88 kDa was 100-fold greater than that of HMW-HA at 683 kDa [7].

HA is a dynamic molecule that can differentially promote or inhibit pathogenesis based on its molecular weight, physicochemical properties, and accessibility to multiple hyaluronan-binding proteins, which are proteins capable of binding to hyaluronan [9,10,11]. Identified receptors of HA include CD44, hyaluronan-mediated motility receptor (RHAMM), lymphatic vessel endothelial hyaluronan receptor (LYVE-1), immunoglobulin-like lectin (SIGLEC-9), Toll-like receptor 2 (TLR2) and TLR4, hyaluronan endocytosis receptor (HARE), cell migration-inducible and hyaluronan-binding protein (CEMIP), and transmembrane protein 2 (TMEM2) [12]. HA molecules and their receptors play various roles in regulating biological processes such as cell migration, lymphocyte homing, signaling, and other immune responses [12,13,14]. HMW-HA can also exhibit anti-inflammatory and analgesic effects on wound healing, as well as on the repair of cutaneous and mucosal tissues, by binding to specific receptors [5,11,15,16,17,18,19,20,21,22,23]. However, due to the high molecular weight of HMW-HA, the binding affinity of HMW-HA to HA receptors on cells embedded in deep tissues is relatively weak [24,25,26].

HMW-HA can be degraded by hyaluronidases to generate products with different molecular sizes, interchangeably referred to as small HA fragments, HA oligosaccharides, or LMW-HA. It is generally believed that LMW-HA possesses potent proinflammatory and proangiogenic properties. An LMW-HA that combines the good tissue permeability of LMW-HA with the biological activity of HMW-HA would be highly valuable in medicinal applications. It has been reported that HA with a molecular weight of 35 kDa, referred to as HA35, which was originally isolated from human colostrum, possesses a unique capability to protect tissues from injury and reduce systemic inflammation without eliciting the various proinflammatory responses typically induced by LMW-HA molecules [27,28,29]. Preclinical data demonstrate that the administration of extraneous HA35 protects the colonic epithelium from bacterium-induced colitis by increasing the expression of antimicrobial β-defensins and tight junction (TJ) proteins. HA35 treatments also reduce intestinal permeability and proinflammatory cytokine release [14,24,25,26].

Using recombinant human hyaluronidase PH20 (rHuPH20) to degrade polymeric HMW-HA with a molecular weight (MW) of 1600 kDa, we produced HA fragments with a specific average molecular weight of 35 kDa [30]. This specifically sized and highly purified HA35, of pharmaceutical grade, has been demonstrated to mimic the protective effects of LMW-HA isolated from human colostrum. However, its bioactivity, biological effects on immune cells, and in vivo biodistribution have yet to be fully characterized. The present study aimed to evaluate the cytological activity of HA35 compared to that of HA1600 in murine and human cells. Using cell culture methods, we determined the effects of HA35 and HA1600 on the cell migration of proinflammatory macrophages, microglia, and lymphocytes. The impact of HA35 on cell migration, specifically related to HA receptor CD44, was further analyzed. We quantitatively characterized the effects of HA35 on the cellular generation of inflammatory biomarkers, including tumor necrosis factor alpha (TNF-α), nitric oxide (NO), reactive oxygen species (ROS), and inducible nitric oxide synthase (iNOS). Additionally, we conducted high-throughput sequencing to compare the differences in cell mRNA expression responses to HA35 and HA1600 treatment, providing a direction for studying the non-tissue permeability-associated mechanisms at the cytological level. Ultimately, we radiolabeled HA35 with ^125^I and ^99m^Tc and performed scintigraphic imaging studies to reveal the in vivo deposition and migration of HA35 administered either systemically or intradermally in C57BL/6J mice. The data from this study lay the foundation for further studies to promote the clinical translation of the HA35 product.

## 2. Materials and Methods

### 2.1. HA35 Production and Molecular Weight Determination

Ultrapure water was first added to the autoclaved fermenter while the temperature was adjusted to 37 °C. Subsequently, MgCl2 and NaCl were added to the fermenter, and sufficient stirring was carried out to achieve final concentrations of 1 mM and 85 mM, respectively. The stirring paddle of the machine was activated once the negative pressure reading on the vacuum gauge reached 0.08–0.09 MPa, and the speed was adjusted to a range of 900–1400 RPM. Pharmaceutical-grade HA1600 (Bloomage Biotech, Jinan, China) at a concentration of 2% was then added and stirred well, followed by the addition of recombinant human hyaluronidase PH20 (rHuPH20) with an initial activity of 20,000 U/g. The mixture was sheared for 4–6 h at 37 °C, maintaining a constant rotation speed of 300 RPM.

Upon the completion of the enzymatic process, the thermostatic water in the machine was emptied, and the steam inlet was opened to raise the temperature to 84–95 °C to inactivate the hyaluronidase. During the enzymatic digestion of HMW-HA, samples of HA35 were collected from the sampling wells at enzymatic digestion times of 5 min and 1, 3, 4, 5, and 6 h. Leech hyaluronidase (HAase), a gift from Dr. Zhen Kang at Jiangnan University, was used as the control for HA enzymatic digestion. The molecular weight was determined using both the agarose gel and gel permeation chromatography multi-angle light scatter methods. All samples were diluted to a final concentration of 5 mg/mL. The diluted samples and standards were mixed 4:1 with loading buffer. Electrophoresis was conducted at 80 V for 1 h, after which the agarose gel was placed in a container containing a solution of Coomassie brilliant blue (CBB) stain [30]. The container was then set on a shaker at a speed of 40–60 rpm to facilitate staining for 2 h. After staining, the gel was destained for another 2 h, until the sample bands became clearly visible, and photographed under bright light. 

Online gel permeation chromatography multi-angle light scattering (GPC-MALS) combined with a refractive index detector (RID) was used to analyze the molecular weight of the samples. High-performance liquid chromatography (HPLC) analysis was performed at 40 °C, using an RID and a Shodex SB-804 HQ gel column (Φ8 mm × 300 mm), with a mobile phase of 0.02% sodium azide, a flow rate of 1 mL/min, and an injection volume of 100 μL. To determine the relative molecular mass of each sample, a specific amount was weighed to prepare a 1 mg/mL polysaccharide solution, subsequently filtered before HPLC analysis. The molecular mass distribution was analyzed by measuring the mass concentration and light scattering intensity at different angles using RID and a laser detector, respectively. The refractive index increment (dn/dc) value was determined as 0.138. The molecular mass distribution of the test samples was obtained using the data processing software ASTRA 8.2 (Wyatt Technology Santa Barbara, CA, USA) [30].

### 2.2. Cell Culture

BV-2 murine microglia cells, RAW264.7 murine macrophages, human H9 T lymphocyte cells, and BALL-1 B lymphocyte cells were commercially purchased from Procell Life Science & Technology Co., Ltd. (Wuhan China). Macrophages were adherently cultured in Dulbecco’s modified Eagle’s medium (DMEM) (Sigma-Aldrich, St. Louis, MO, USA), and the BV-2 murine microglia cells were also adherently cultured, but in minimum essential medium (MEM) (Sigma-Aldrich, USA). The H9 and BALL-1 cells were suspension cultured in Roswell Park Memorial Institute Medium (RPMI 1640) (Sigma-Aldrich, USA). All cells were cultured and supplemented with 10% FBS (Zhejiang Tianhang Biotechnology Co., Ltd., Tongxiang City, Zhejiang, China), along with 100 units/mL of penicillin and 100 μg/mL of streptomycin (HyClone, Logan, UT, USA). The culture was maintained at 37 °C in a 5% CO_2_ environment. The medium was changed every 3 days. Cells were passaged upon reaching 80% confluency. These cells were then treated with lipopolysaccharide (LPS) (Sigma-Aldrich, USA), or HA as described below and subsequently used for protein extraction and preparation.

### 2.3. Cell Migration Assays

After two passages, RAW264.7, BV2, BALL-1, and H9 cells were harvested, and the cell density was adjusted to 3 × 10^8^ cells/mL using their respective culture media. A 0.8% agarose gel was prepared and sterilized under high pressure. This gel was mixed with cell culture media containing 20% FBS and 1% penicillin/streptomycin in a 1:1 volume ratio. These mixtures were then incubated with the respective cell suspensions in a 1:1 volume ratio and placed in a 37 °C water bath for insulation. A 2 μL volume of the cell–agarose mixture was transferred to the center of each well in a 96-well plate using a pipette, forming a cell drop with a 2 mm diameter, which was then left to solidify at 4 °C. After 15 min of solidification, 100 μL of culture medium containing various treatment agents was added to the wells and cultured at 37 °C for 72 h. The treatments included (1) 300 μg/mL HA35, (2) 300 μg/mL HA1600, (3) 1 nM fMLP, (4) 10 μg/mL anti-CD44 antibody (Abcam, Cambridge, UK), (5) 300 μg/mL HA35 + 10 μg/mL anti-CD44 antibody, and (6) 300 μg/mL HA1600 + 10 μg/mL anti-CD44 antibody. Four parallel testing repetitions were performed for each treatment. The cell preparations were visualized under an inverted phase-contrast microscope at 4× magnification. The radius of the agarose gel droplet was used as a baseline to measure the area at the furthest edge of cell migration from the droplet. ImageJ 1.53k software (NIH, Bethesda, MD, USA) was used to calculate the area difference, representing the migration distance.

### 2.4. Measurement of Cell TNF-α and NO Production

The effects of HA35 and HA1600 on TNF-α and NO production were compared in macrophages, microglia, and lymphocytes. Four groups of RAW264.7 cells and BV2 cells were each treated with 500 µg/mL LPS, 300 µg/mL HA35, 300 µg/mL HA1600, and PBS for 72 h, respectively. The levels of TNF-α in the supernatants from RAW264.7 and BV2 cell cultures at 72 h post-treatment were measured using an ELISA DY2.10 kit (R&D Systems, Minneapolis, MN, USA). The TNF-α levels in both cell types were reported as the concentration in the supernatant (ng/mL). Supernatants aspirated from cultures of RAW264.7 cells, BV2 cells, BALL-1 cells, and H9 cells were analyzed for NO levels using an NO assay kit (S0021S) (Beyotime, Shanghai, China) after 72 h of culture. The optical density of the reactions in the microplate wells was measured using a microplate spectrophotometer set to dual wavelengths of 450 nm and 520 nm [31].

### 2.5. Measurement of Cellular Reactive Oxygen Species Generation

RAW264.7 cells and BV2 cells, which had been centrifuged and adjusted to a cell density of 3 × 10^6^ cells/mL in 200 μL of media, were seeded in 24-well plates. A fluorescent probe, Dihydrorhodamine-123 (DHR-123), for measuring ROS production was added to each well at a final concentration of 5 µM. The cells were then incubated in the dark at 37 °C for 15 min. Subsequently, treatments including 10% FBS, 300 μg/mL HA35 + 10% FBS, and 300 μg/mL HA1600 + 10% FBS were added to wells. The cells were stimulated with 500 ng/mL LPS and incubated for 30 min. Green fluorescence was measured using a flow cytometer with a 488 nm wavelength laser for excitation and the detection of emission at 575 nm. The average fluorescence intensity was labeled as M1, and the total fluorescence intensity was calculated as the product of % gated and the mean fluorescence intensity (MFI).

### 2.6. RNA Sequencing

To further investigate the role of HA35 in regulating gene expression levels in immune cells, RNA sequencing was conducted on RAW264.7 cells and BV2 cells to identify transcriptional changes associated with HA production regulation. RAW264.7 cells and BV2 cells were inoculated in 6-well plates at a density of 1 × 10^6^ cells/mL and cultured overnight. After adhesion, the cells were replaced with serum-free DMEM and MEM, and then resuspended in serum-free RPMI 1640. One milliliter of 300 µg/mL HA35 or HA1600 was added to the respective culture systems and incubated at 37 °C for 6 h. Post-incubation, the cells were detached using a cell scraper and transferred to RNase-free EP tubes. RNA was extracted using 500 µL of TRIzol solution per sample to prepare for transcriptome sequencing. The extracted total RNA samples were analyzed using 1% agarose gel electrophoresis, a Nanodrop 2000 spectrophotometer, a Qubit^®^ 3.0 Fluorometer, and an Agilent 2100 Bioanalyzer (all from Thermo Fisher Scientific, Waltham, MA, USA). Following transcriptome sequencing, library preparation, and quality verification, sequencing was performed primarily using the Illumina HiSeqTM X TEN system [32,33,34].

### 2.7. Radiolabeling of HA35

HA35 was radiolabeled with Iodine-125 (^125^I) and Technetium-99m (^99m^Tc) to produce ^125^I-HA35 and ^99m^Tc-HA35 for assessing the in vivo biodistribution of HA35 using the methods described previously [35]. HA35 was activated with CNBr and then iodinated with ^125^I via tyramine (TA) substitution. In brief, 10 mg of HA35 in 1 mL of water was activated by adding 200 µL of CNBr (40 mg/mL). The mixture was adjusted to pH 11 and maintained for 5 min by adding 0.2 M NaOH. The activated HA35 was separated from the reaction mixture on a small Sephadex G25 (PD10) column equilibrated with 0.2 M borate buffer pH 8.0. CNBr-activated HA35 molecules were incubated overnight with 0.5–1.0 mg TA. A Sephadex G-25 column separated TA-bound HA35 (TA-HA35) from unbound TA. Iodination was carried out by incubating 250 µL of TA-HA35 with 1.0 mCi ^125^I in Pierce precoated iodination tubes for 15 min at room temperature. Reaction mixtures were removed from the tubes to terminate iodide oxidation and then loaded into PD10 columns for purification. The radiolabeling yield and radiochemical purity (RCP) of the labeled product, ^125^I-HA35, were determined via instant thin-layer chromatography (ITLC) using a 50/50 mixture of 0.1 M sodium citrate solution at pH 5 and acetonitrile as the mobile phase. ^125^I-HA35 with an RCP greater than 96% was stored at 2–8 °C for use within two weeks.

HA35 was directly labeled with ^99m^Tc by reducing ^99m^Tc using stannous chloride (SnCl_2_) to generate Tc(V) as [TcOCl_4_]^−^, which then bound to the carboxyl group of HA35, forming a stable compound of ^99m^Tc-HA35. In brief, physiological saline solution and 0.1 N HCl were prepared and bubbled with N2 for 15 min. A volume of 5 μL of SnCl_2_ (2 mg/mL) was added to a mixture of 1.0 mg of HA35 in 20 μL of water and 250 μL of ^99m^TcO4- (~10 mCi) in saline. The reaction mixtures were incubated at 80 °C for 90 min. Size-exclusion high-performance liquid chromatography (SEC-HPLC) analysis was performed to measure the radiolabeling yield and RCP of the ^99m^Tc-labeled product, using a Shodex KW 802.5 column (Thomson Instrument Co., Oceanside, CA, USA) equilibrated and eluted with PBS at pH 7.4, at a flow rate of 0.8 mL/minute. ^99m^Tc-HA35 with an RCP greater than 96% was used for animal imaging studies within 6 h post-labeling.

The stability of the radiolabeled products was evaluated in fresh rat serum at 37 °C. A solution of purified ^125^I-HA35 or ^99m^Tc-HA35 was mixed with serum at a 1:1 volume-to-volume (*v*/*v*) ratio. The RCP of the mixture was selectively analyzed over 5 h of post-mixing, using ITLC for ^125^I-HA35 and SEC-HPLC for ^99m^Tc-HA35.

### 2.8. Imaging ^125^I-HA35 and ^99m^Tc-HA35 Biodistribution in Healthy Mice

A dedicated, custom-built small animal imager, the iQID camera [35], was used to image the biodistribution of radiolabeled HA35 in healthy C57BL/6J mice. Five mice, aged 6–8 weeks, were used for lymphatic imaging with ^125^I-HA35. Mice 1 and 5 were intradermally injected with 20–25 μCi ^125^I-HA35 into all four paws, while Mice 2–4 received a 20–25 μCi ^125^I-HA35 injection into the hind paws. Whole-body iQID images of Mouse 1 were repeatedly collected with 5 min acquisitions at 1–5, 6–10, 26–30, and 176–180 min post-injection. Mice 2–5 were imaged at 180 min post-injection. Each mouse was anesthetized with 1–2% isoflurane and placed directly on the iQID camera for imaging acquisition. At the end of the imaging session, the mouse was euthanized, and tissue biodistribution measurements were taken to determine the percent injected dose per gram of tissue (%ID/g).

^99m^Tc-HA35 (0.8–1.2 mCi) was intravenously injected in six C57BL/6J mice, aged 6–8 weeks. Among the six mice, one mouse was imaged with the iQID camera at 30 min, 3 h, and 21 h after the injection of ^99m^Tc-HA35 to assess biodistribution changes related to free ^99m^Tc radioactivity released from the ^99m^Tc-labeled HA backbone, due to the potential instability of ^99m^Tc-HA35. The other 5 mice were imaged only at 3 h post-injection. Each mouse was anesthetized with 1–2% isoflurane and placed directly on the iQID camera for whole-body imaging, with an acquisition time of 15 min. The datasets of iQID images were processed using the public-domain AMIDE 1.04 software (SourceForge.net (accessed on 1 November 2023)). Subsequently, each mouse was euthanized. Blood and major organs were harvested, weighed, and subjected to radioactivity measurements. Biodistribution results in the samples were calculated and expressed as %ID/g.

### 2.9. Data Processing and Statistical Analysis

All quantitative data are expressed as the mean ± standard deviation (SD). Statistical tests were performed using GraphPad Prism software (Version 9.3.1, San Diego, CA, USA). The results between two groups were compared using an unpaired Student’s *t*-test. A probability value (*p*) >0.05 was considered not statistically significant and marked as ‘ns’; *p* < 0.05 was considered statistically significant and marked as “*”; *p* < 0.01 was considered more statistically significant and marked as “**”; and *p* < 0.001 was considered highly statistically significant and marked as “***”.

## 3. Results

### 3.1. HA35 Production

**Figure 1:**
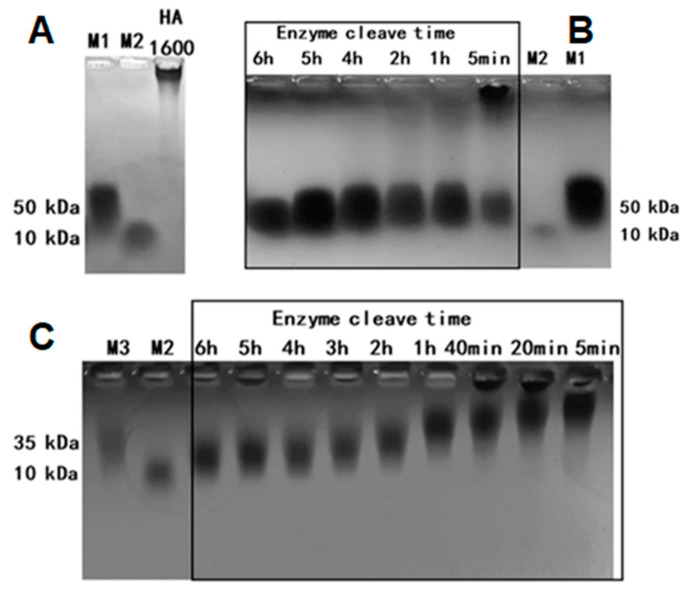
Representative gel electrophoresis of the molecular weight distribution of HA35 and HA1600. (**A**) HA1600; (**B**) the HA fragments when HA1600 was cleaved by the rhuPH20 enzyme for 6, 5, 4, 2, and 1 h, and 5 min; (**C**) the HA fragments when HA1600 was cleaved by the leech hyaluronidase for 6, 5, 4, 3, 2, and 1 h, and 40, 20, and 5 min. M1, M2 and M3: the marker of a 50 kDa, 10 kDa and 35 kDa molecular weight.

With rHuPH20 at a final concentration of 20,000 U/g, HMW HA1600 was successfully degraded to the LMW fragment HA35. The results of the molecular weight distribution of HA35 and HA1600 were determined via gel electrophoresis and are shown in Figure 1. The band corresponding to HA1600 was well clustered at the location of the spot sample due to its large molecular weight (Figure 1A). Changes in HA molecular weight were observed after 5 min of enzymatic digestion (Figure 1B). A lower-molecular-weight band resulting from the enzymatic digestion of HA1600 for 1 h became visible at 35 kDa. The outcomes of enzymatic digestion performed for 2, 4, 5, and 6 h indicated that rHuPH20, at a final concentration of 20,000 U/g, could stably produce HA35, regardless of the duration of the enzymatic reaction. Compared with Figure 1C, the molecular weight of HA digested by leech hyaluronidase decreased gradually from 5 min to 6 h. This result shows that the enzymokinetics of rHuPH20 are markedly different from those of the traditional enzyme. Using the GPC-MALLS method, the specific molecular weights of HA fragments resulting from the 6 h cleavage of HA1600 are presented in Table 1. The average molecular weight of HA fragments was determined to be 35 kDa, consistent with the results obtained from agarose gel electrophoresis.

### 3.2. HA35- and HA1600-Regulated Immune Cell Migration

**Figure 2:**
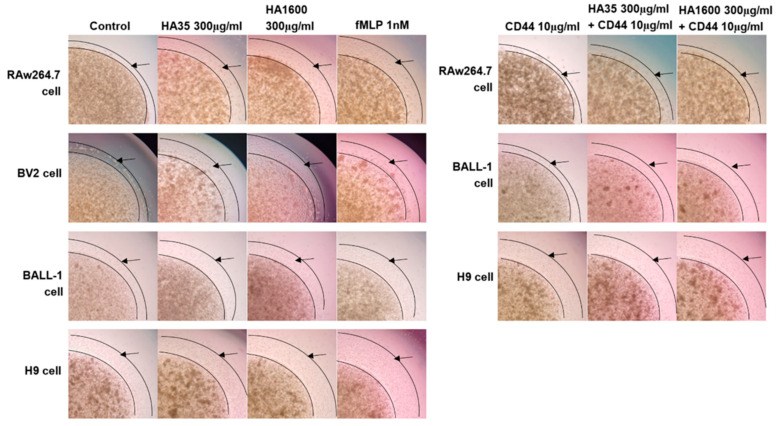
Representative microphotographs (120×) illustrating the effects of HA35 and HA1600 on the migration behavior of various immune cells, including macrophages (RAW264.7), microglia (BV2), B lymphocytes (BALL-1), and T lymphocytes (H9), in comparison with saline as the negative control and fMLP as the positive control. Bright-field images capture areas showing the longest cell migration distances. The leading edge, indicating the migration distance, is marked by arrows in the images.

**Figure 3:**
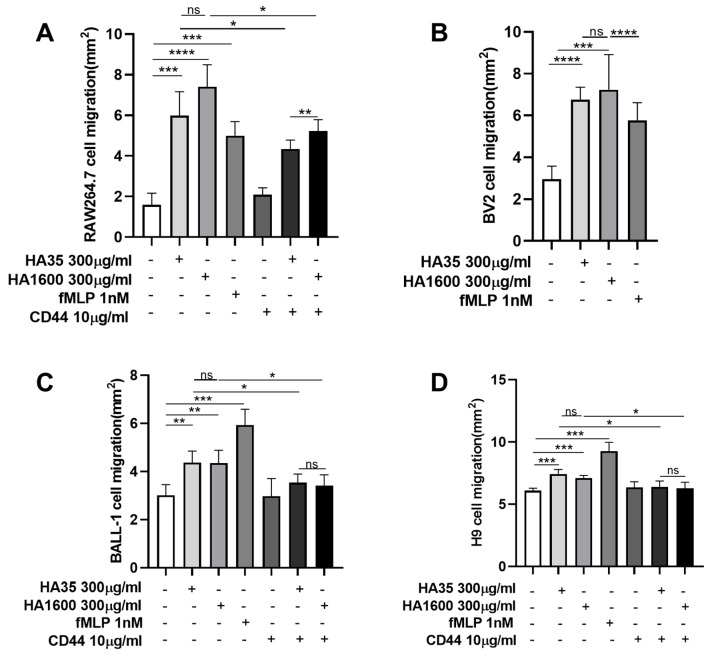
Quantitative analysis of cell migration, determined by measuring the area encompassed by cell migration from the innermost point of the cell-containing droplet to the furthest edge of cell movement into the surrounding culture medium. The results are presented for each cell type, RAW264.7 cells (**A**), BV2 cells (**B**), BALL-1 cells (**C**), and H9 cells (**D**), with four replicates (*n* = 4) for each group. ns: no significant (*p* > 0.05), * *p* < 0.05, ** *p* < 0.01, *** *p* < 0.001, and **** *p* < 0.0001 for comparison between two groups.

With agarose gel simulating the in vivo interstitial matrix and chemokine fMLP as a positive control, we observed and compared the HA35- and HA1600-regulated migration of RAW264.7, BV2, BALL-1, and H9 cells, as depicted in Figure 2 and Figure 3. These cells were incubated with HA35 or HA1600 for 72 h. HA35 demonstrated similar effects on the migration modes of macrophages, microglia, and lymphocytes with no statistical difference compared to HA1600 (*p* > 0.05) in all cells. When RAW264.7 cells, BALL-1 cells, and H9 cells were treated with anti-mouse and anti-human CD44 antibodies (10 µg/mL) in the presence of HA35 or HA1600, cell migration was significantly inhibited compared to that of cells treated solely with HA35 or HA1600 (*p* < 0.05 in all pairs), as illustrated in Figure 2 and Figure 3. The application of the CD44 antibody alone did not affect cell migration. These findings suggest that the immune cell migration stimulated by HA35 and HA1600 is mediated via the HA–CD44 interaction on the inflammatory cells, following a similar mechanism. Additional cell migration data are available in the Supplemental Data.

### 3.3. HA35 and HA1600 Effects on TNF-α and NO Production of Immune Cells

**Figure 4:**
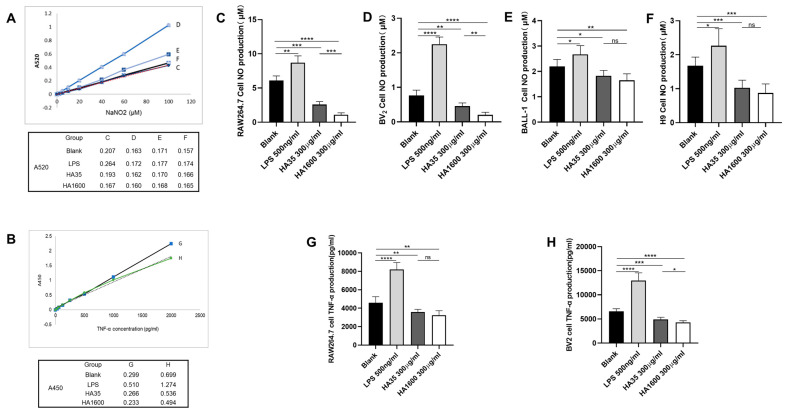
Results of NO generation during the migration of RAW264.7 cells, BV2 cells, BALL-1 cells, and H9 cells, as well as TNF-α production during the migration of RAW264.7 cells and BV2 cells, (n = 4 for each group). Notes: Panels (**A**,**B**) illustrate the standard curve for NO and TNF-α quantification, and the corresponding absorbance readings, respectively. Panels (**C**–**F**) depict the NO content secreted during the migration of the aforementioned cells. Panels (**G**,**H**) reveal the amount of TNF-α secreted during the migration of RAW264.7 and BV2 cells. Significance levels are indicated as ns: no significant (*p* > 0.05) * *p* < 0.05, ** *p* < 0.01, *** *p* < 0.001, and **** *p* < 0.0001 for comparisons between two groups.

With cells treated with LPS (500 ng/mL) serving as the positive control, the production of TNF-α and NO in RAW264.7 and BV2 cells was significantly reduced by both HA35 and HA1600, as demonstrated in Figure 4. HA35 and HA1600 also downregulated NO generation in BALL-1 and H9 cells. Overall, HA35 and HA1600 exhibited similar effects in downregulating TNF-α and NO production in macrophages, microglia, and lymphocytes in cell culture studies. However, HA1600 showed a more pronounced effect in reducing NO generation in RAW264.7 cells (*p* < 0.001) and BV2 cells (*p* < 0.01) compared to HA35.

### 3.4. HA35 and HA1600 Effects on ROS Production of Macrophages and Microglia

**Figure 5:**
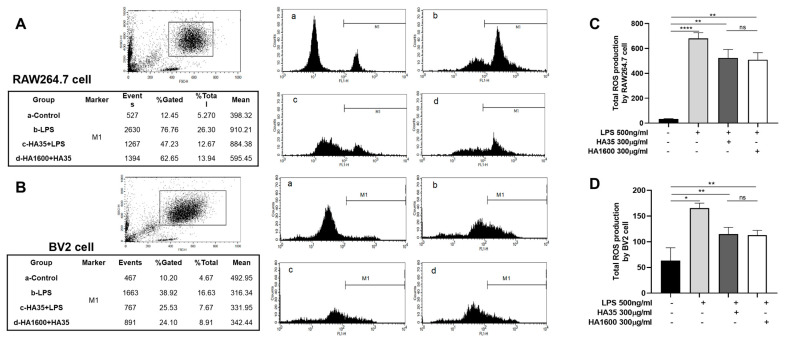
Flow cytometric results showing ROS production in RAW264.7 macrophages and BV2 microglia in response to saline control, HA35, and HA1600 treatments. Notes: (**A**,**C**) RAW264.7 cells; (**B**,**D**) BV2 cells. Values are presented as the means ± SDs (n = 4 for each group). ns: no significant (*p* > 0.05), * *p* < 0.05, ** *p* < 0.01, and **** *p* < 0.0001 for comparison between two groups.

We utilized LPS-activated RAW264.7 macrophages and BV2 microglia to compare the effects of HA35 and HA1600 on the ROS production of immune cells. As illustrated in Figure 5, the cell populations in diagrams A and B display two peaks. The peak representing inactivated cells is less than 1 × 10^2^, while the peak for activated cells exceeds 1 × 10^2^. Upon stimulation by influencing factors, the peak for activated cells shifts gradually to the right. M1 denotes the fluorescence intensity of all activated cells, with higher values on the X-axis indicating stronger fluorescence. The flow cytometric results shown in Figure 5 reveal that LPS (10 ng/mL) significantly increased ROS production in RAW264.7 cells (*p* < 0.001) and BV2 cells (*p* < 0.05). Both HA35 and HA1600 significantly reduced LPS-induced ROS in RAW264.7 cells (HA35 vs. control, *p* < 0.0001; HA1600 vs. control, *p* < 0.01) and BV2 cells (HA35 vs. control, *p* < 0.05; HA1600 vs. control, *p* < 0.01). No significant differences were observed in ROS production between HA35 and HA1600 in either RAW264.7 cells (*p* > 0.05) or BV2 cells (*p* > 0.05).

### 3.5. RNA Sequencing Results of HA35 and HA1600 Regulating Gene Expression

**Figure 6:**
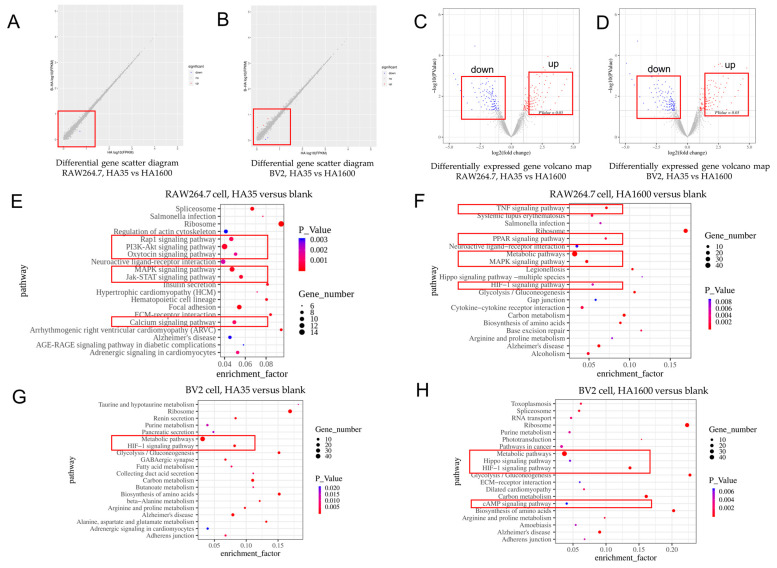
RNA seq analysis of HA35 versus HA1600 for differentially expressed genes in RAW264.7 cells and BV2 cells. (**A**,**C**) HA35 versus HA1600 for RAW264.7 cells; (**B**,**D**) HA35 versus HA1600 for BV2 cells; (**E**) RAW264.7 cells, HA35 versus blank; (**F**) RAW264.7 cells, HA1600 versus blank; (**G**) BV2 cells, HA35 versus blank; (**H**) BV2 cells, HA1600 versus blank. A higher number of off-diagonal points suggests a more significant difference in gene expression between the two samples. Points in the volcano plot that are farther from the origin on the x-axis indicate a more significant variation in the gene expression between samples. Larger values on the y-axis indicate the greater significance of differentially expressed genes.

The RNA-Seq scatter plots demonstrate that the gene expression levels in RAW264.7 and BV2 cells treated with either HA35 or HA1600 were overall similar, as depicted in Figure 6A,B. The number of genes regulated by HA35 and HA1600 was significantly larger in BV2 cells than in RAW264.7 cells. Figure 6C,D utilize volcano plots to illustrate the distribution of differentially expressed genes (DEGs) in RAW264.7 and BV2 cells treated with HA35 and HA1600, respectively. The criteria for screening DEGs were set as a log2 fold change (L2FC) greater than 1, with a *p*-value of less than 0.05. Table 2 details the number of DEGs relative to the blank control. The gene expression profile changes induced by HA35 and HA1600 in both RAW264.7 and BV2 cells exhibited a high degree of similarity.

The SPAM1 genes, encoding hyaluronidase PH20, were significantly upregulated in the RAW264.7 macrophages (Table 3) and BV2 microglia (Table 4) treated with either HA35 or HA1600. Both HA35 and HA1600 consistently regulated the inflammatory genes Tnfrsf4 and Tnfrsf6 in RAW264.7 cells, significantly suppressing the expression of Tnfrsf4 and promoting the expression of Tnfrsf6 compared to the blank control (*p* < 0.05). Proteins encoded by these genes are members of the TNF-receptor superfamily, with Tnfrsf4 known to activate the NF-κB pathway, regulating pro-inflammatory cytokine production, leukocyte recruitment, and other adaptive immune functions. Both HA1600 and HA35 significantly increased the expression of immune regulatory gene IL-1, with HA1600 showing a more pronounced effect than HA35 (*p* < 0.01). The expression of IL-27 was upregulated by both HA35 and HA1600 (*p* < 0.05). HA35 slightly increased IRF6 expression in BV2 cells (*p* < 0.05), while HA1600 decreased it (*p* < 0.01). There were subtle differences between HA35 and HA1600 in regulating cytokine and chemokine expression genes. In RAW264.7 cells, HA35 upregulated the gene expression of the pro-inflammatory cytokine IL17D (*p* < 0.05), whereas HA1600 showed no significant change compared to the control (*p* > 0.05). In the regulation of the chemokine genes CCL7 and CCL22, HA1600 demonstrated a stronger regulatory capacity than that of HA35 (*p* < 0.05). In BV2 cells, HA35 and HA1600 differed in their effects on IL-10 (*p* < 0.001) and IL-33 (*p* < 0.01).

As shown in Figure 6, the KEGG pathway analysis of the RNA-seq gene expression method revealed that 70 pathways were enriched in the HA35 group of RAW264.7 cells compared to the number of those in the blank control (*p* < 0.05). Among them, there were seven pathways relating to inflammatory signaling pathways, including MAPK, PI3K-Akt, Rap1, HIF-1, Ras, Hippo, and AMPK (Figure 6E). Relative to the blank control, the HA1600-treated group showed enrichment in 36 pathways (*p* < 0.05), with 5 related to inflammation signaling pathways, including TNF, MAPK, HIF-1, Hippo, and PI3K-Akt (Figure 6F). Overall, HA35 affected a greater number of immune-related pathways in RAW264.7 cells compared to HA1600. Additionally, the DEGs in the BV2 cells treated with HA35 were enriched in 38 pathways (HA35 vs. control, *p* < 0.05), including 2 inflammation signaling pathways related to HIF-1 and MAPK signaling, similar to those observed in RAW264.7 cells (Figure 6G). In the HA1600 group of BV2 cells, 47 pathways were enriched in total (HA1600 vs. control, *p* < 0.05), among which three, the HIF-1, cAMP, and PI3K-Akt signaling pathways (Figure 6H), were associated with inflammation. Thus, the results of the RNA-seq gene expression analysis in RAW264.7 and BV2 cells indicate that HA35 and HA1600 have a similar trend in regulating immune cells but present some differences in their regulatory pathways.

### 3.6. Imaging of Radiolabeled HA35 Biodistribution in Living Mice

The ^125^I-iodination of HA35 generated a 70–75% labeling yield of ^125^I-HA35 as determined via ITLC analysis. After gel filtration, the RCP of the ^125^I-HA35 product exceeded 96%. The direct ^99m^Tc-labeling protocol consistently generated an over 95% yield of ^99m^Tc-HA35. After purification, ^99m^Tc-HA35 with an RCP of ≥98% was obtained for biodistribution imaging studies. We have noted that our current protocols for radiolabeling HA fragments with ^125^I or ^99m^Tc are impractical for labeling HA1600, due to its poor solubility and the lack of suitable purification methods.

Using ITLC analysis, stability tests of ^125^I-HA35 in serum at 37 °C for 5 h revealed that the degradation was 3.5 ± 0.4% (n = 4). The results of SEC-HPLC analysis demonstrated the good thermodynamic stability of ^99m^Tc-HA. After incubation in serum at 37 °C for 5-h, the RCP (%) of ^99m^Tc-HA35 was 95.8 ± 3.2 (n = 4).

**Figure 7:**
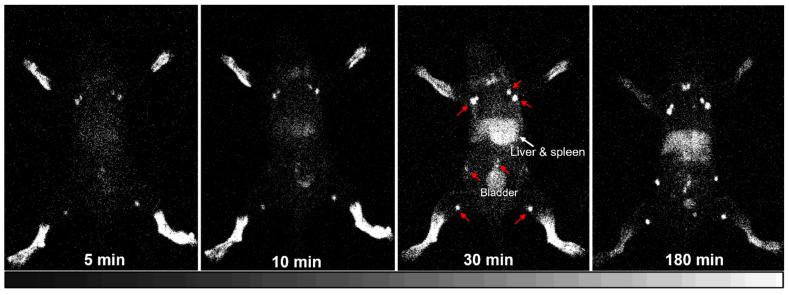
Representative series of mouse iQID images of ^125^I-HA35 collected at 5, 10, 30, and 180 min post-intradermal injections of the four paws. The image at each time point was generated via the 5 min acquisition of radioactive counts. The fast lymphatic circulation of ^125^I-HA35 was initially observed at 5 min. The lymph nodes exhibited the highest level of radioactive uptake (counts per pixel) compared to the other organs at 180 min post-injection. As marked by red arrows on a 30 min image, popliteal, inguinal, axillary, brachial, and mandibular lymph nodes were superiorly visualized. Lumbar, iliac, sacral, pancreaticoduodenal, and cervical lymph nodes were also visible. Along with the radioactivity being decreased in the footpad, the radioactive intensity was reversely increased in the lymph nodes, spleen, and liver.

**Figure 8:**
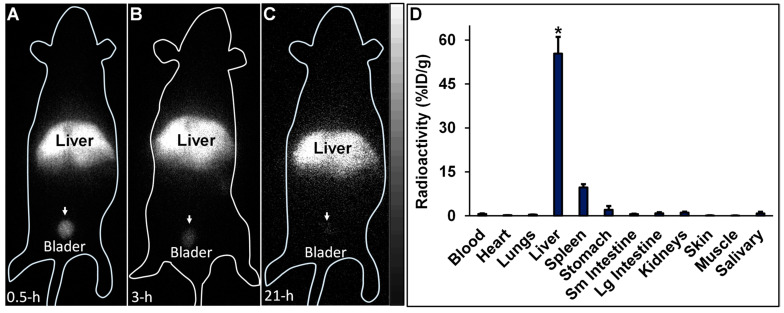
(**A**–**C**) Representative in vivo iQID images of ^99m^Tc-HA35 (1.0 mCi) at 0.5 (**A**), 3 (**B**), and 21 h (**C**) post-injection in a C57BL/6J mouse. Prominent radioactive uptake of ^99m^Tc-HA35 was observed in the liver. (**D**) Results of the postmortem biodistribution measurements in 5 mice euthanized 3 h after the IV injection of ^99m^Tc-HA35. * *p* < 0.0001 for liver radioactivity compared to other tissues.

The iQID gamma-ray imager was demonstrated with superior utility for the imaging of radiolabeled HA35 biodistribution as presented in Figure 7 and Figure 8. With intradermal injections into the mouse footpads, ^125^I-HA35 entered the lymphatic circulation and accumulated in the lymph nodes within 5 min, followed by uptake into lymphatic organs (spleen) within 10 min, with persistent retention in the lymph nodes, spleen, and liver. As shown in Figure 7, popliteal lymphatic drainage and lymph node mapping in mice were clearly revealed on planar iQID images. ^125^I-HA35 iQID imaging enabled the rapid localization of popliteal, inguinal, axillary, brachial, mandibular, lumbar, iliac, sacral, pancreaticoduodenal, and cervical lymph nodes. Typically, the popliteal, inguinal, axillary, and brachial lymph nodes exhibited a higher uptake of ^125^I-HA35 compared to other lymph nodes and major organs, such as the liver, kidneys, and bladder, likely due to their direct and shorter lymph drainage routes. Using ROI analysis, the average radioactive intensity (randomized unit per pixel) in all visible lymph nodes and the liver was 5.83 ± 0.96 versus 2.94 ± 0.62 with no significant difference (*p* > 0.05) at 180 min post-injection, which was significantly higher than the radioactive distribution in the soft tissue background (0.12 ± 0.03) (*p* < 0.001). The radioactive intensity in the popliteal, inguinal, axillary, and brachial lymph nodes was significantly higher than that in the liver (9.58 ± 1.81 vs. 2.94 ± 0.62, *p* = 0.0262). Radioactive uptake in the thyroid gland was possibly caused by a small amount of free iodine (^125^I) resulting from the detached labels of HA35. Additional lymphatic images of ^125^I-HA35 in the other four mice are available in the Supplemental Data.

iQID images of ^99m^Tc-HA35 biodistribution in mice, acquired at 0.5, 3, and 21 h post-intravenous injection, are shown in Figure 8. At 0.5 and 3 h after the injection of ^99m^Tc-HA35, the radioactive distribution was predominantly localized in the liver. The spleen was less visible due to its overlap with the liver in the planar images. A moderate amount of radioactivity was observed in the bladder. Twenty-one hours later, the predominant radioactivity still remained in the liver. The kidneys were either invisible or less visible, although minimal radioactivity was still detectable in the bladder. No clearly detectable radioactive accumulation from free ^99m^Tc pertechnetate was observed in the salivary glands, stomach, or thyroid. The in vivo findings obtained via iQID imaging were confirmed by the ex vivo biodistribution measurements, summarized in Figure 8D. These measurements were collected from five other mice euthanized 3 h after ^99m^Tc-HA35 injection. The 3 h iQID images of ^99m^Tc-HA35 in these five mice are available in the Supplemental Data.

## 4. Discussion

The chemical compositions of LMW-HA and HMW-HA are identical. However, the length of the polymer chains results in different molecular weights and marked differences in HA signaling, biological effects, and tissue permeability [22,36,37,38,39,40]. Studies using bioengineered HA fragments have provided new insights into the functions of HA in maintaining cellular microenvironment homeostasis and in the process of inflammation and wound healing. Our previous studies demonstrated the use of recombinant human hyaluronidase PH20 for the stable production of HA35 with an average molecular weight of 35 kDa through the cleavage of HA1600 [30]. In vitro experiments revealed that HA35 can pass through 220 nm pore-size filter membranes without resistance, unlike the parent HA1600. This current study utilized an in vitro cell culture method and demonstrated that HA35 and HA1600 molecules interact with cells in the culture medium without restrictions on tissue permeability due to molecular size. We compared the effects of HA35 and HA1600 on the migration of immune cells, including the impact on the migration and dissemination of macrophages, microglia, and lymphocytes. These effects are related to the expression of the HA receptors CD44 and LYVE-1, as well as to the levels of the inflammatory marker NO.

HA35 may play a beneficial role in regulating the cell-mediated immune response to tissue injury and infection. During inflammation-mediated tissue injuries, various inflammatory cells, such as neutrophils, macrophages, and microglia, are recruited to the site of injury, primarily from the bloodstream and bone marrow. Activated lymphocytes from lymph nodes migrate to the site of injury or infection, release inflammatory factors, and exert immune functions. Our previous in vitro studies revealed that HA35 fragments are capable of promoting the migration of freshly isolated human mononuclear cells, including leukocytes and monocytes [30]. The delivery of extraneous HA35 to the inflammatory site may influence the migration and activity of various immune cells, including neutrophils, macrophages, dendritic cells, and lymphocytes (such as T cells, B cells, and NK cells). Thus, by stimulating the migration and proliferation of various cell types involved in wound healing, such as fibroblasts and endothelial cells, HA35 may accelerate tissue repair and regeneration. HA35 may also promote angiogenesis, improving the supply of oxygen and nutrients to the injured tissue and facilitating the healing process.

As partially evident in this study, the effects of HA35 on inflammation and tissue repair involve interactions with various cell surface receptors, such as CD44 and Toll-like receptors (TLRs). These interactions modulate cell migration, proliferation, and inflammatory cellular responses, which are crucial for wound healing and immune regulation. CD44 is a transmembrane glycoprotein that plays a pivotal role in these biological processes. In the context of inflammatory injury, CD44 becomes particularly significant. It is widely recognized as a key receptor in the metabolism of HA. We therefore selected CD44 as the primary HA receptor in this study to explore the role of HA35 in regulating immune cell migration. We demonstrated that the stimulation of inflammatory cell migration by HA35 is mediated through its interaction with CD44 receptors on the cell surface, similarly to HA1600. The enhanced cell migration effects of HA35 and HA1600 in macrophages and lymphocytes could be inhibited by CD44 antibodies.

Our data from this study demonstrated that HA35, similar to HA1600, could inhibit the production of the inflammatory marker NO. Furthermore, we found that both HA35 and HA1600 also reduced ROS production in activated immune cells. ROS are key signaling molecules that play a crucial role in the progression of inflammatory disorders. Enhanced ROS generation at the site of inflammation can cause endothelial dysfunction and tissue injury. The generation of ROS in phagocytes, such as neutrophils and macrophages, is linked to the activation of the NADPH oxidase complex, while NO production is controlled by inducible nitric oxide synthase (iNOS) in response to inflammation. The potential mechanism through which HA35 inhibits the production of NO and ROS from inflammatory cells may involve various cellular pathways. HA35 may interact with receptors like CD44 and TLRs on immune cells, affecting nitric oxide synthase (NOS), especially iNOS, that can modulate the production of NO. This interaction can decrease pro-inflammatory cytokines (TNF-α, IL-1β, IFN-γ), indirectly reducing iNOS expression and NO production. The binding of HA35 to CD44 and TLRs may modulate downstream signaling pathways that affect the activation of the NADPH oxidase complex in phagocytic cells to suppress ROS production. This could involve altering the phosphorylation state of components of the NADPH oxidase complex or affecting the translocation of these components to the cell membrane where they become active. In addition, HA35 could modulate inflammatory cytokine pathways related to ROS generation and act as an antioxidant, scavenging ROS or enhancing endogenous antioxidant enzymes.

This study utilized high-throughput mRNA sequencing to investigate the effects of HA35 and HA1600 on gene expression in specific inflammatory cell types. It is believed that LMW-HA fragments, produced during tissue injury or inflammation, act as signaling molecules, promoting cytokine and chemokine production based on the tissue environment, immune cell types, and the size and concentration of HA fragments. In this study, we identified several commonalities between HA35 and HA1600 in modulating immune signaling pathways. Notably, both HA35 and HA1600 were found to upregulate IL-1 and IL-27 expression in RAW264.7 macrophages and BV-2 microglial cells. In addition, HA35 and HA1600 similarly influenced the expression of the inflammatory genes Tnfrsf4 and Tnfrsf6 in RAW264.7 cells. Despite these parallels, variations were observed in the specific types of immune cell gene expression affected by each agent, suggesting that HA35 and HA1600 have additional, distinct roles in the regulation of immune cell molecular pathways. The HA35 products generated by rHuPH20 enzymatic cleavage in this study exhibit similar regulatory features in cytokine and chemokine production as observed in rHuPH20-produced LMW-HA fragments reported previously [41,42]. Further studies are necessary to validate these RNA-Seq findings.

In our lymphatic imaging studies utilizing ^125^I-HA35, we observed that the HA35 fragment demonstrates effective dermal penetration and superior tissue permeability. These characteristics are likely attributable to its relatively smaller molecular size, high mobility, and low viscosity. Following intradermal ^125^I-HA35 administration, rapid radioactive accumulation was observed in the lymph nodes within 5 min. By 10 min, significant radioactivity was detected in the spleen. It is uncertain whether these processes are primarily driven by passive diffusion or involve protein/receptor-mediated transport mechanisms [23]. Upon entering the interstitial fluid, HA35 fragments may interact with proteins and other molecules in the extracellular matrix to form HA protein and glycocalyx complexes, which are then transported to the lymphatic system. The mechanisms underlying the rapid lymphatic drainage and systemic absorption of this specific-size HA fragment remain to be fully elucidated. 

To the best of our knowledge, this study represents the first innovative exploration of lymphatic circulation using ^125^I-iodinated HA fragments and the iQID imaging technique. However, imaging with ^125^I-HA cannot be performed using a conventional gamma camera or SPECT imager due to the lower-energy gamma photons emitted by ^125^I. Concerns regarding the long half-life of ^125^I (60 days) and risk of contamination also limit the utility of ^125^I-HA35. After initially applying ^125^I-HA35 in our lymphatic imaging project with healthy mice, we developed a rapid protocol for the direct ^99m^Tc-labeling of HA fragments (^99m^Tc-HA), which replaced ^125^I-labeling. We then conducted ^99m^Tc-HA lymphatic imaging studies using various mouse models (data not presented here). In comparison with ^125^I, ^99m^Tc is widely used in clinical imaging in nuclear medicine and offers advantages such as low cost, optimal emission energy, and a short half-life (6.04 h).

Following the intravenous administration of ^99m^Tc-HA35, radioactivity was predominantly localized in the liver, with a lesser amount observed in the spleen. Importantly, no detectable radioactivity was visualized in the lungs via iQID imaging, indicating that the ^99m^Tc-labeled HA35 fragments did not form microaggregates trapped in the pulmonary microvasculature. Moderate radioactivity was observed in the bladder, indicative of renal excretion, but there was no significant radioactive uptake in the salivary gland and stomach. Typically, excessive uptake in the salivary gland and stomach is associated with free ^99m^Tc pertechnetate, a decomposition product of ^99m^Tc-labeled molecules. The biodistribution of radiolabeled HA35 in the liver, lymph nodes, and spleen observed in this study was comparable to that of other HA fragments administered subcutaneously or intravenously, as reported in several studies [36,37,38,39,40]. 

HA, particularly its high-molecular-weight-form (HMW-HA), shows potential for improving lifespan, with potential applications in cancer and inflammation resistance [8,17,18]. However, these potential benefits are still under investigation, and further research is warranted. Exogenously administered HMW-HA has the potential to restore damaged tissue, making it a potential therapeutic strategy in inflammation-associated diseases. However, HMW-HA exhibits limited tissue permeability, posing challenges to elucidating its true functions in vivo and effectively utilizing it in therapeutic applications. In this study, we observed that HA35 and HA1600 exhibited similar biological activities in various cell culture models, where tissue permeability was not a factor. Both HA35 and HA1600 appeared to facilitate the diffusion and homing of immune cells, such as macrophages and lymphocytes, suggesting a potential new therapeutic pathway for various human diseases. Our preliminary clinical trials have yielded promising results, indicating that the tissue-permeable HA35 may be effective in pain relief and wound healing (see Clinical Trials NCT05756595 and NCT05764226 at ClinicalTrials.gov (accessed on 26 December 2023)). More comprehensive studies are necessary to fully understand the implications of HA35 in medical treatments.

There are several limitations in this study. First, the permeability of HA35 and HA1600 were not directly compared via in vivo tissue analyses. In our previous and current experimental preparations, HA35 and HA1600 were compared through filtrations using filters with a pore size of 220 nanometers. The results indicate that HA35 has better permeability than HA1600. When prepared at concentrations greater than 0.2%, HA1600 formed gel-like substances and could not pass through a 220-nanometer filter. In contrast, HA35 consistently flowed freely through this filter size. It is known that particles smaller than 220 nanometers, typically referred to as nanoparticles, are tissue-permeable. Second, the biological activities of HA35 and HA1600 in regulating cell behaviors and functions were not completely consistent in our studies, as revealed by RNA-seq gene expression analysis. These gene expression results, which relate to cellular immune function, were obtained preliminarily from RAW264.7 macrophages and BV2 microglia under cell culture conditions. The RNA-seq gene expression data in these two types of immune cells may not fully reveal the roles of HA fragments in the innate immune response during inflammation. Third, this study obtained a significant amount of in vitro bioactivity data to compare HA35 with HA1600, but it lacks in vivo comparative biodistribution data to fully understand their biological differences due to unavailable radiolabeled HA1600 for imaging studies.

## 5. Conclusions

HA35 and HA1600 exhibit a high degree of similarity in modulating the cellular activities of immune cells, including enhancing immune cell migration and reducing pro-inflammatory mediators such as NO and ROS. These cellular effects are possibly mediated through mechanisms involving binding to HA receptors, such as CD44 and TLRs. Both HA35 and HA1600 demonstrate the ability to modulate gene expression profiles in diverse immune cells, influencing cell signaling pathways and biological functions. While they share a similar trend in the regulation of immune cells, HA35 and HA1600 also exhibit some distinct differences in their influence on inflammatory molecular pathways. Interestingly, HA35 appears to have a more extensive impact on gene regulation, exerting additional and unique effects on a broader range of genes within both human and murine immune cells, compared to HA1600. Upon intradermal or intravenous administration, HA35 fragments can exhibit rapid accumulation in the liver, spleen, and lymph nodes. The rapid lymphatic circulation of HA35 may facilitate water transport to the lymphatic system and enhance immune cell migration to lymph nodes. Therefore, as a promising candidate for the treatment of inflammatory diseases, HA35 may offer a biological capability in inhibiting adverse inflammatory responses in tissue sites with injury or infection, comparable to HMW-HA, while also providing favorable tissue permeability and bioavailability. Scintigraphic imaging using radiolabeled HA35 presents a practical method for studying the biodistribution and metabolism of HA fragments and for evaluating therapies aimed at inhibiting inflammation.

## Figures and Tables

**Table 1:** Molecular weight determination of HA35 and HA1600 via the GPC-MALLS method.

Molar Mass Moments (g/mol)	Hyaluronidase	Time	Mean ± SD	N
HA1600 (kDa)	-	-	1646.67 ± 420.38	3
HA35 (kDa)	rhuPH20 enzyme	6 h	34.61 ± 1.32	3
HA (kDa)	leech hyaluronidase	6 h	25.24 ± 4.18	3

**Table 2:** Number of genes with altered expression levels in cells.

	RAW264.7 Cells		BV2 Cells	
Gene Expression	HA35	HA1600	HA35	HA1600
Upregulated	385	355	419	430
Downregulated	380	397	322	344
Total	765	752	741	774

**Table 3:** Gene expression levels (FPKM) of inflammation-related factors of RAW264.7 macrophages.

Gene	HA35	HA1600	Ctr	*p* (HA35 vs. Ctr)	*p* (HA1600 vs. Ctr)	*p* (HA35 vs. HA1600)
SPAM1	0.0765	0.1265	0.0197	0.0215	0.0010	0.0021
IL-1β	1.8493	0.9824	3.6117	0.0498	0.0001	0.0011
IL-36G	0.0247	0.0184	0.0794	0.0482	0.0207	0.0198
Tnfrsf4	0	0	0.0220	0.0217	0.0217	>0.05
Tnfrsf6	0.0157	0.0157	0	0.0444	0.0444	>0.05
IL17D	0.02389	0.1436	0.0869	0.0472	>0.05	0.0025
LIF	1.1661	0.5303	1.9094	>0.05	0.0001	0.0039
CCL22	0.3623	0.1654	0.3780	>0.05	0.0110	0.0161
CCL7	0.4689	0.2030	0.6678	>0.05	0.0015	0.0316

FPKM stands for fragments per kilobase of transcript per million mapped reads. Ctr: blank control.

**Table 4:** Gene expression levels (FPKM) of inflammation-related factors secreted of BV2 microglia.

Gene	HA35	HA1600	Ctr	*p* (HA35 vs. Ctr)	*p* (HA1600 vs. Ctr)	*p* (HA35 vs. HA1600)
SPAM1	0.116 5	0.156 5	0.029 7	0.0075	0.0010	>0.05
IL-27	0.1268	0.1259	0.3109	0.0286	0.0275	>0.05
INF-zata	0.0000	0.0000	0.0271	0.0214	0.0214	>0.05
IRF6	0.0072	0.0192	0.0000	0.0448	0.0014	>0.05
Tnfrsf13c	0.0050	0.0000	0.0200	>0.05	0.0212	>0.05
Tnfrsf9	0.0282	0.0419	0.0093	>0.05	0.0428	>0.05
Il10	0.0076	0.1661	0.0227	>0.05	0.0005	0.0001
Il33	0.0548	0.0077	0.0078	0.0040	>0.05	0.0039
Ifnb1	0.1861	0.0527	0.1322	>0.05	>0.05	0.0310

## Data Availability

The data presented in this study are available in the article and supplementary material.

## Supplementary Materials

The following supporting information can be downloaded at: https://www.mdpi.com/article/10.3390/life14010097/s1, manuscript-supplementary.
