# Supplementary material for: Characterization, Bioactivity, and Biodistribution of 35 kDa Hyaluronan Fragment
Source: Life (Basel). 2024 Jan 8;14(1):97. doi: 10.3390/life14010097 (PMC10817694; doi:10.3390/life14010097)
Supplement: Supplementary file 1 [file life-14-00097-s001.zip › life-2711444-supplementary.pdf]

Supplemental Data I:

Figure S1A-S1Y. HA35- and HA1600-regulated immune cell migration.

Microphotographs (120X) illustrating the effects of HA35 and HA1600 on the migration behavior of various immune cells, including macrophages (RAW264.7), microglia (BV2), B lymphocytes (BALL-1), and T lymphocytes (H9), in comparison with saline as the negative blank control and fMLP as the positive control. Bright-field images capture areas showing the longest cell migration distances. The leading edge represents the migration distance.

Figure S1A. **Ball-1 Cells, Blank Control**

Plate 1

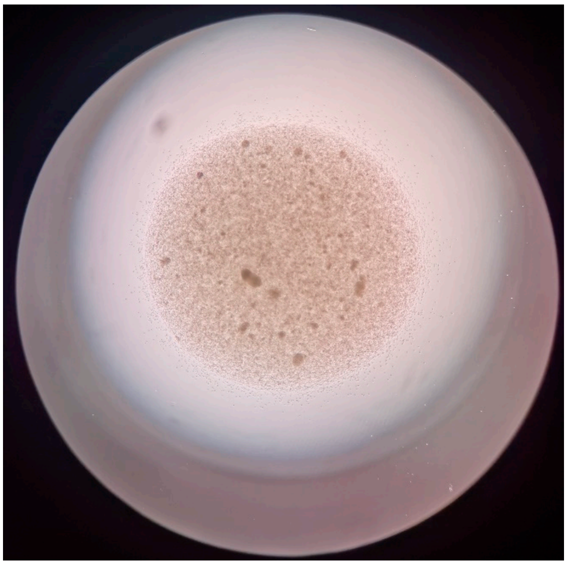

Plate 3

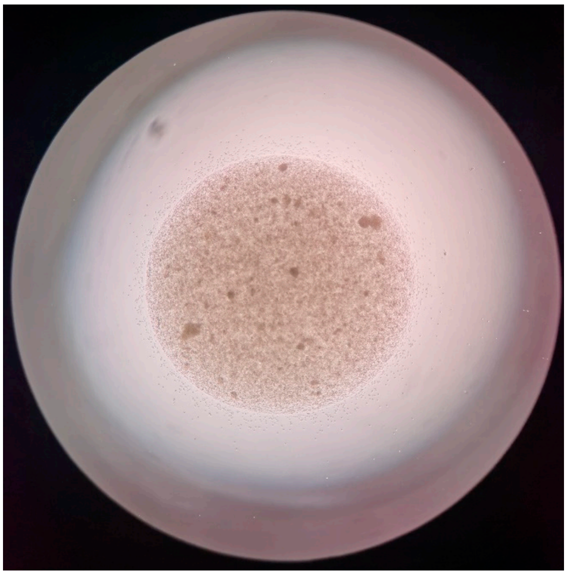

Plate 2

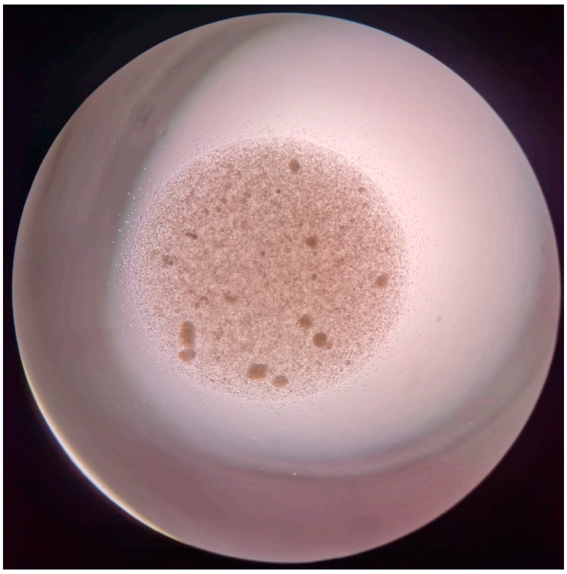

Plate 4

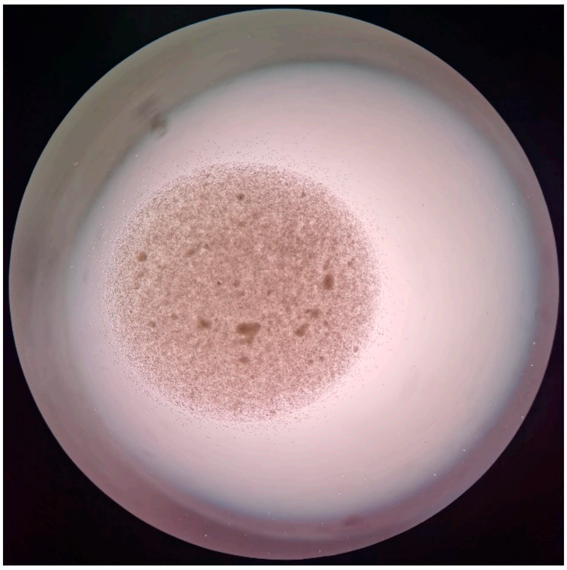

Figure S1B. **Ball-1 Cells with CD44**

Study 1

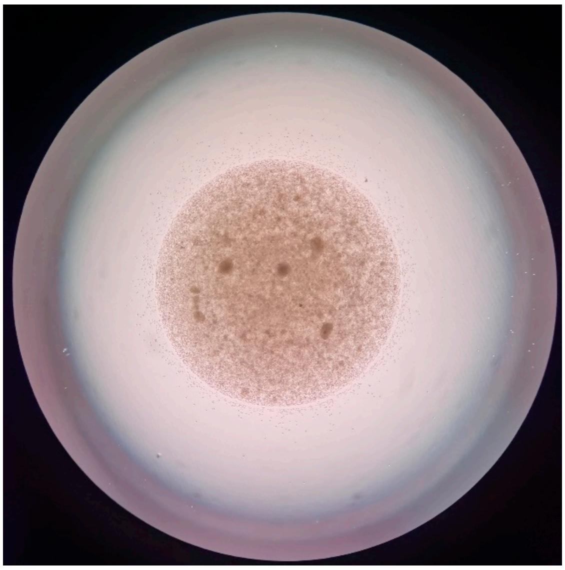

Plate 3

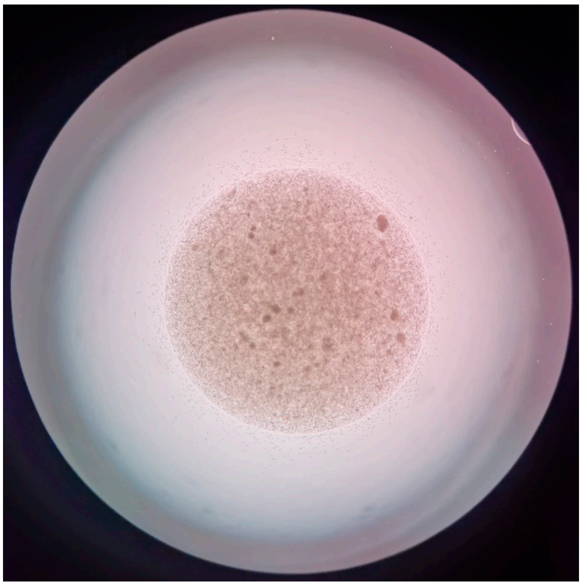

Plate 2

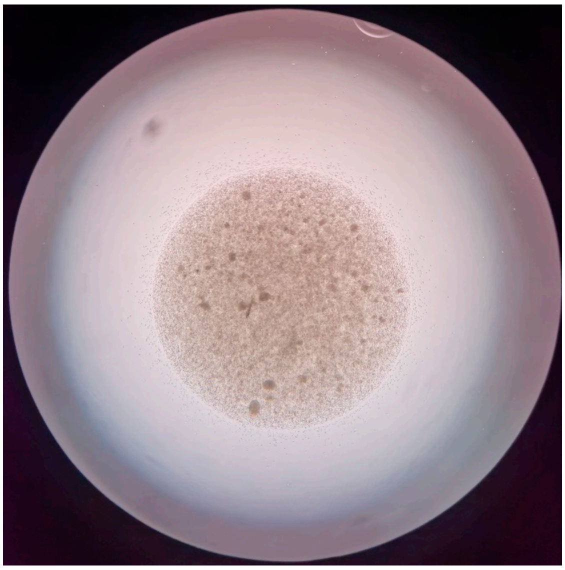

Plate 4

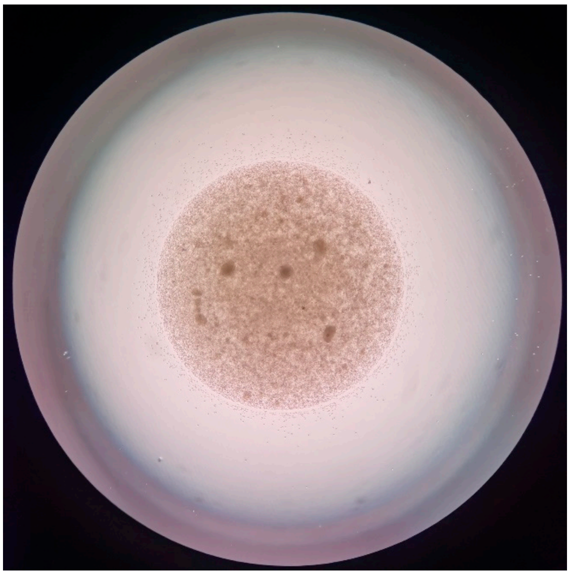

Figure S1C. **Ball-1 Cells with fMLP**

Study 1

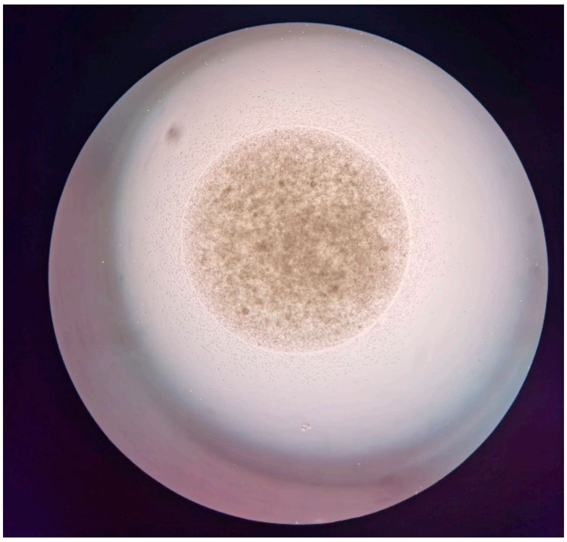

Plate 3

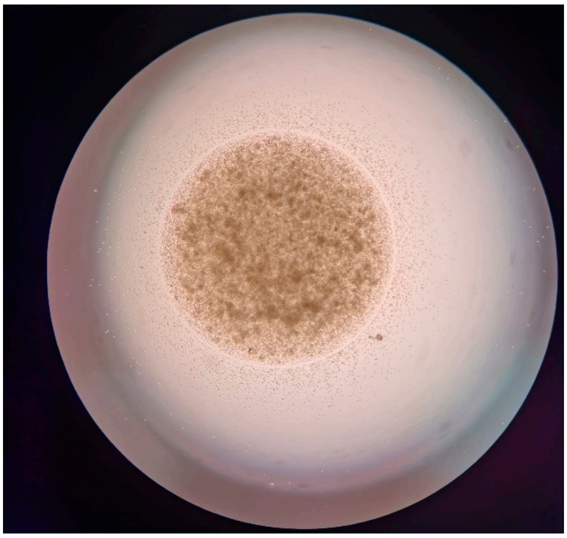

Plate 2

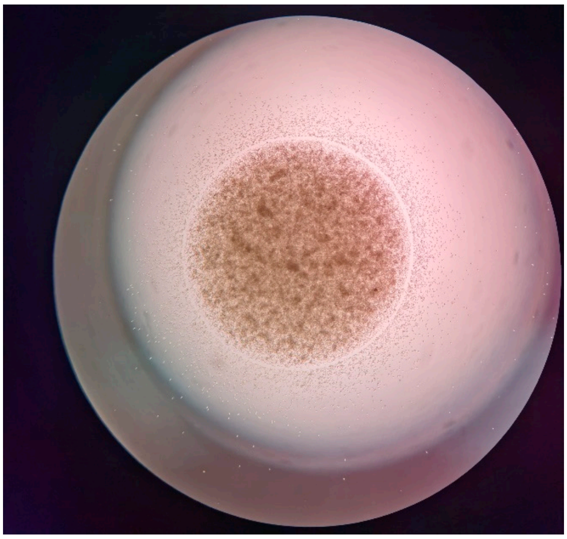

Plate 4

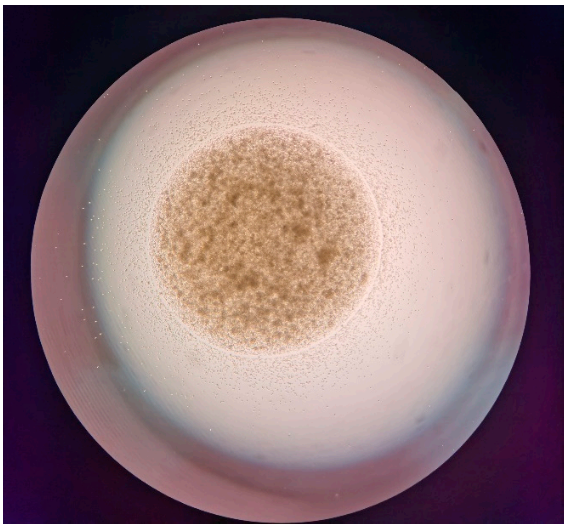

Figure S1D. **Ball-1 Cells with HA35**

Study 1

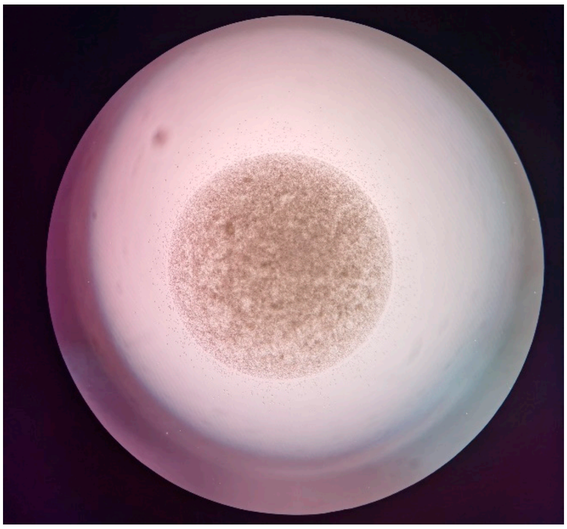

Plate 3

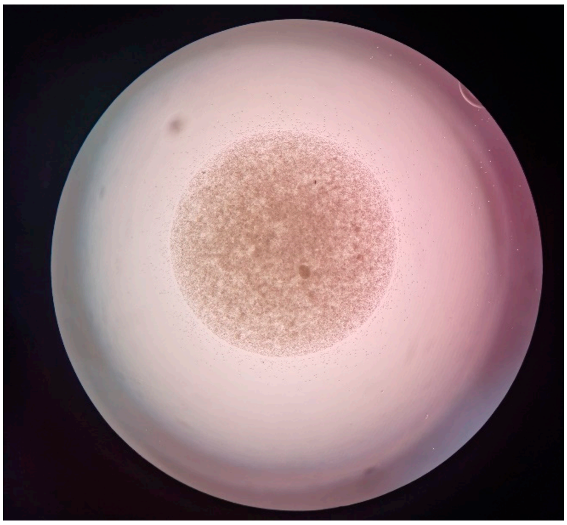

Plate 2

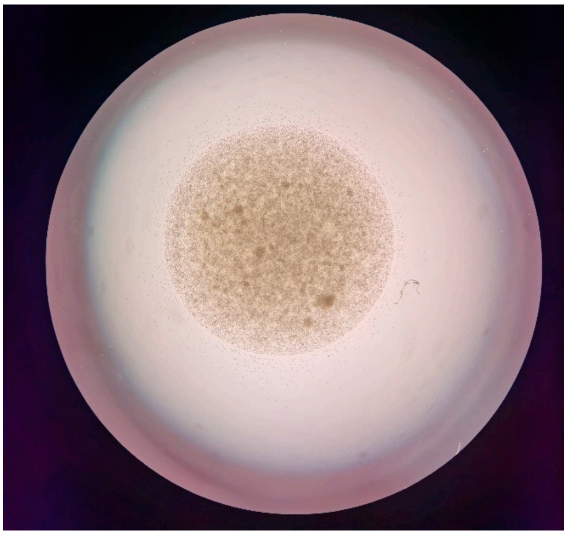

Plate 4

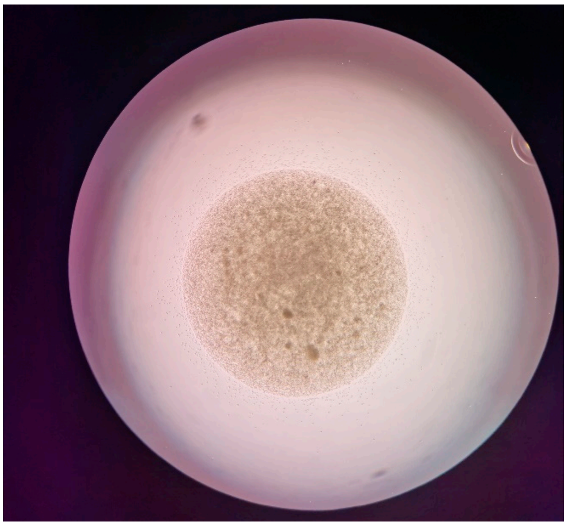

Figure S1E. Ball-1 Cells with HA35 + Anti-CD44

Plate 1

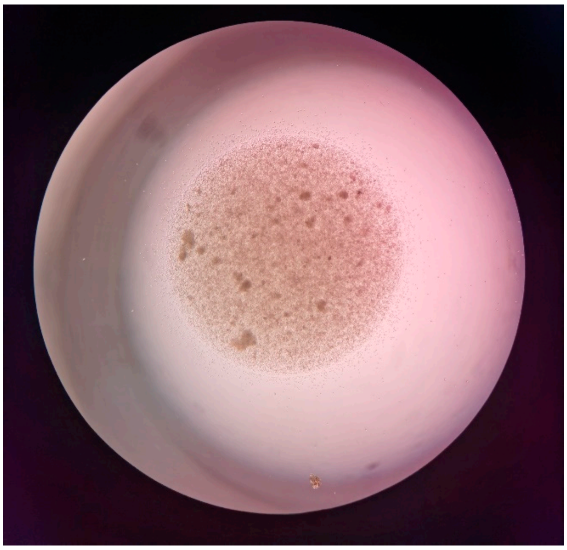

Plate 3

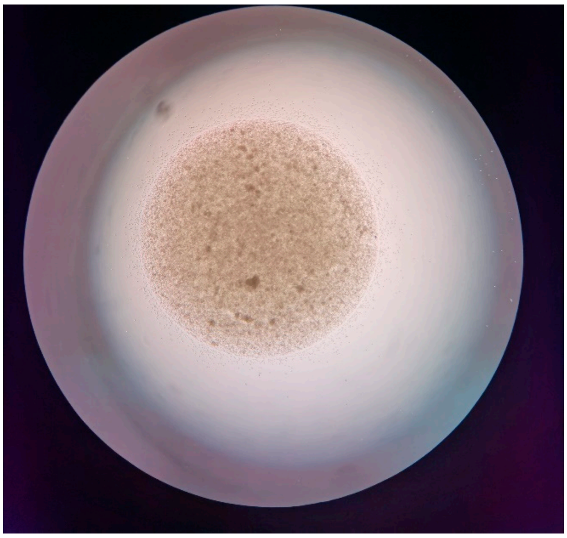

Plate 2

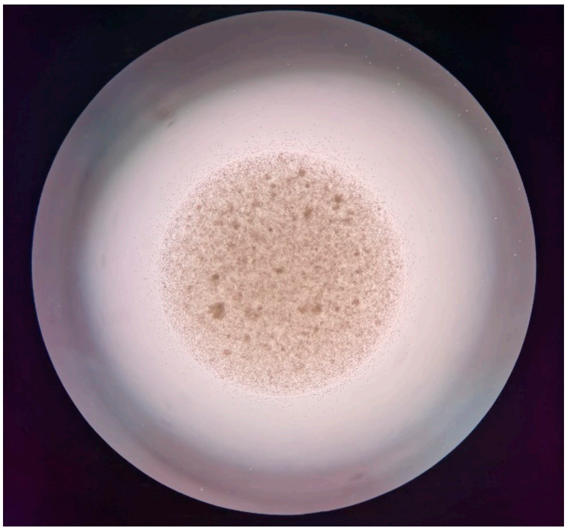

Plate 4

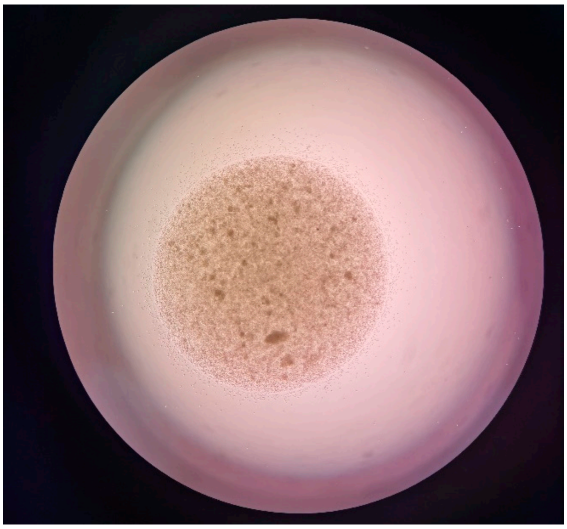

Figure S1F. **Ball-1 Cells with HA1600**

Plate 1

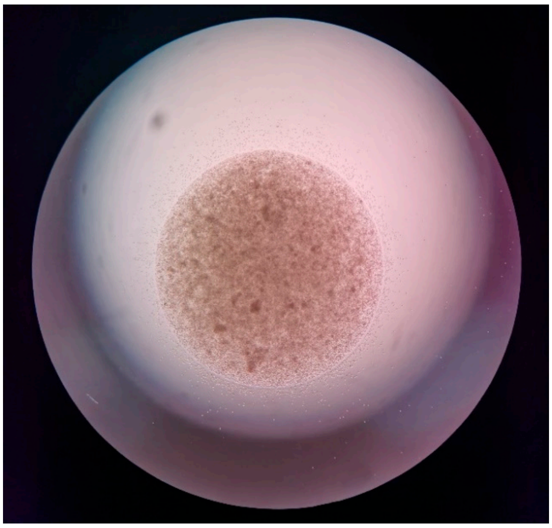

Plate 3

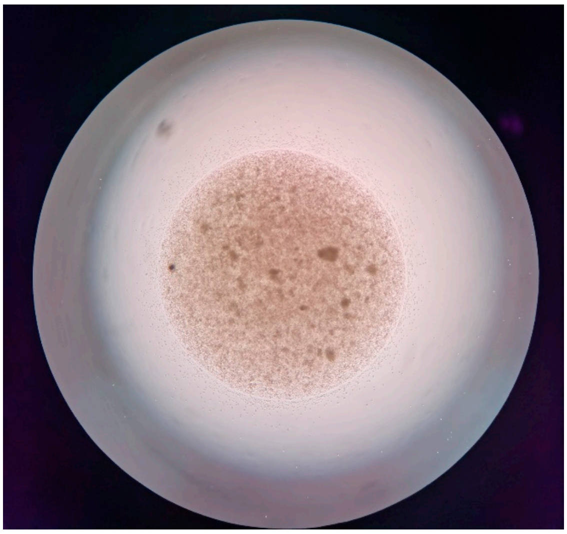

Plate 2

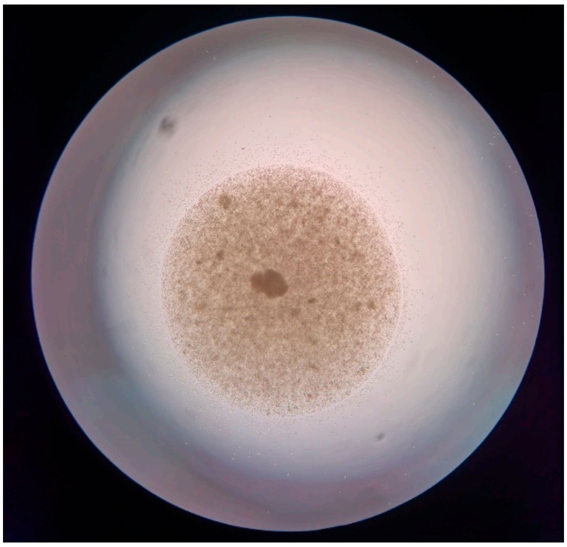

Plate 4

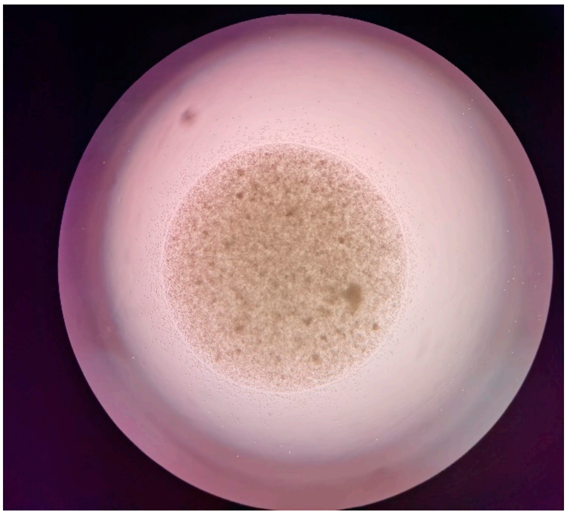

Figure S1G. **Ball-1 Cells with HA1600 + Anti-CD44**

Plate 1

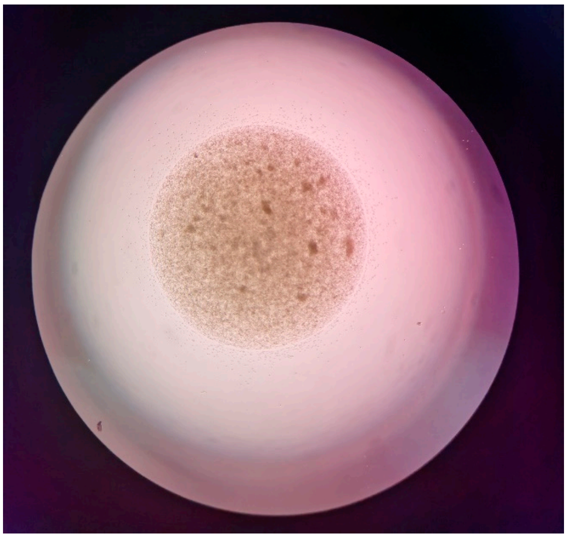

Plate 3

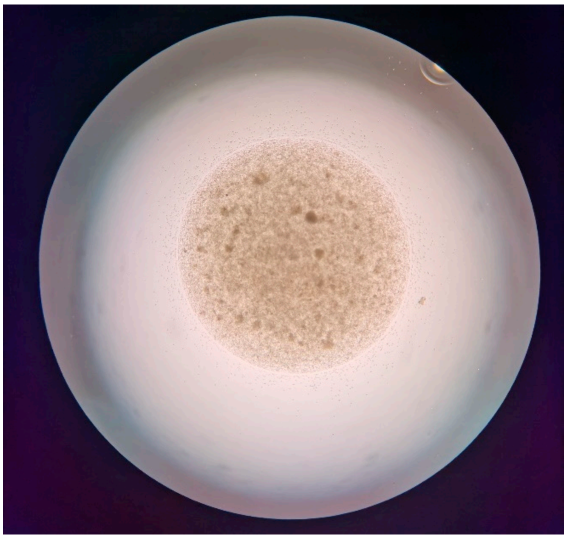

Plate 2

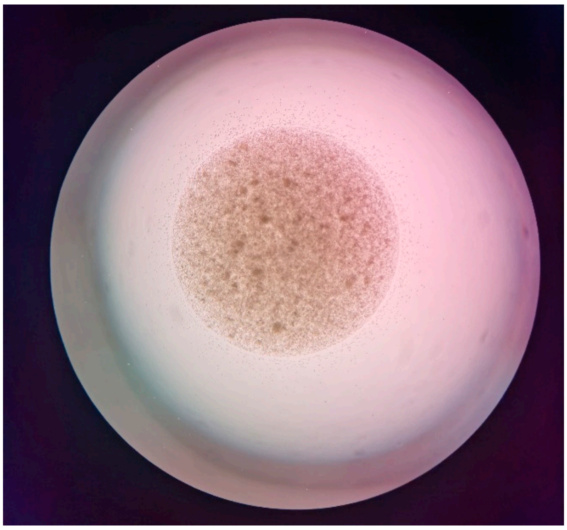

Plate 4

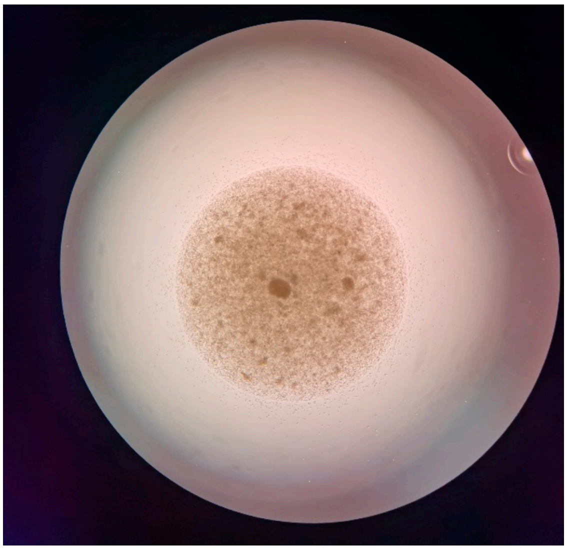

Figure S1H. **BV2 Blank Control**

Plate 1

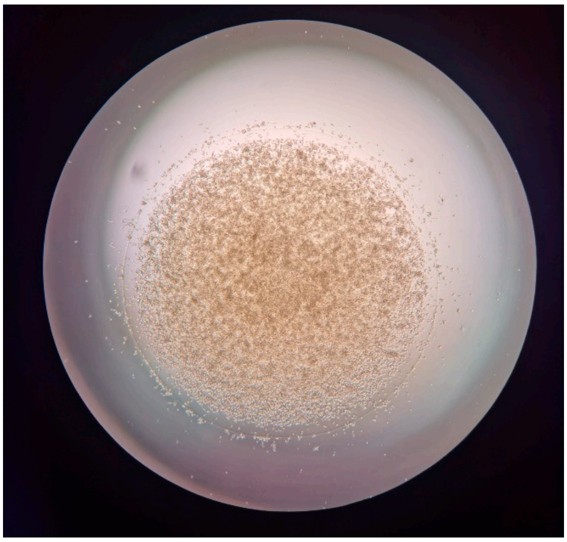

Plate 2

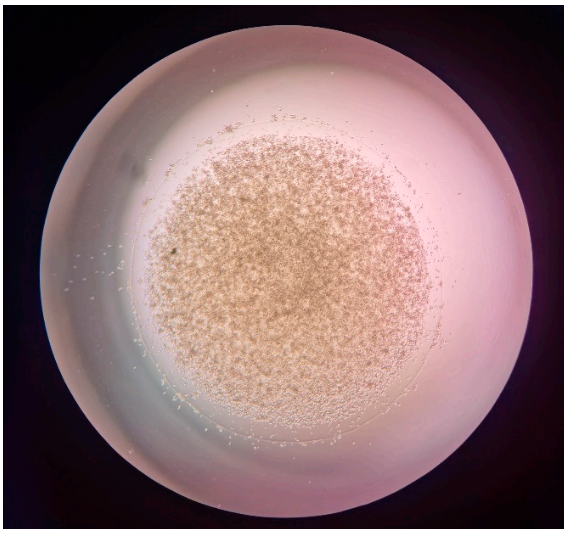

Plate 3

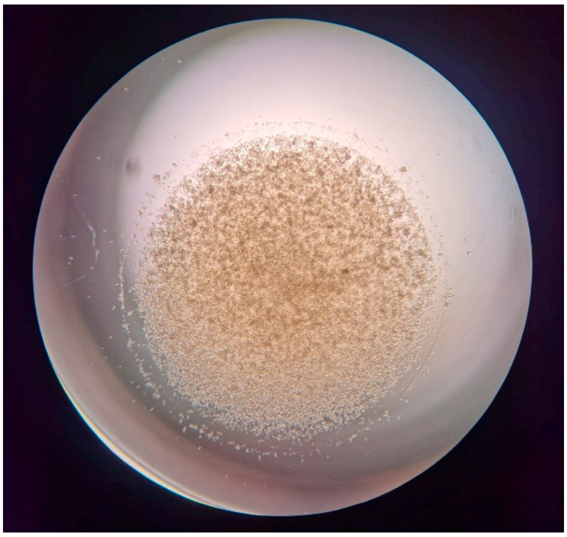

Plate 4

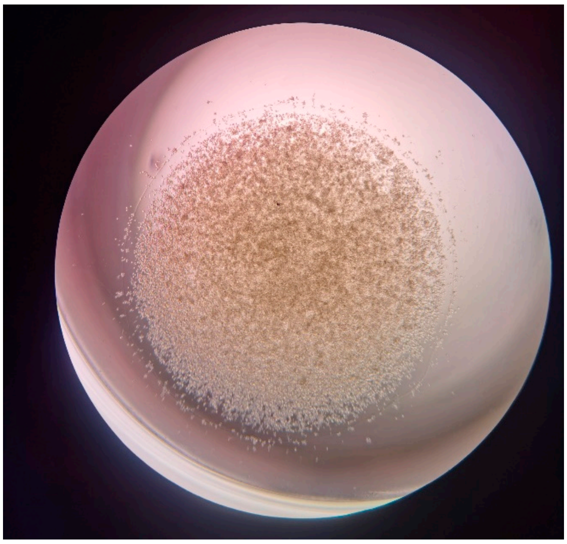

Figure S1I. **BV2 with fMLP**

Plate 1

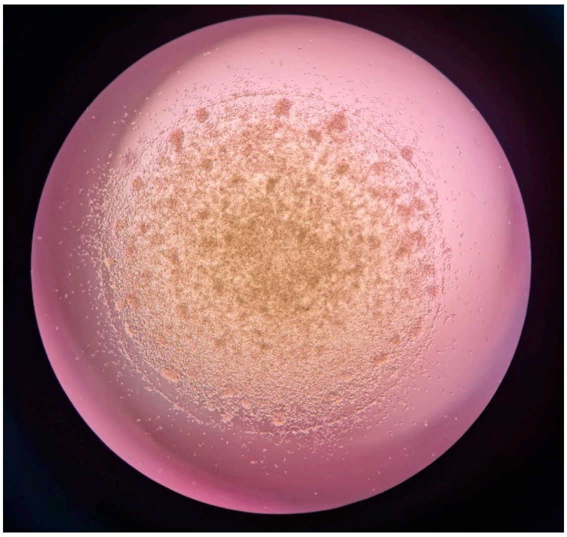

Plate 3

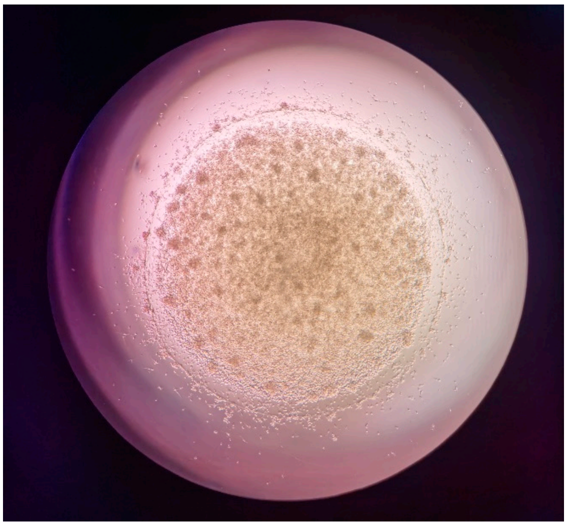

Plate 2

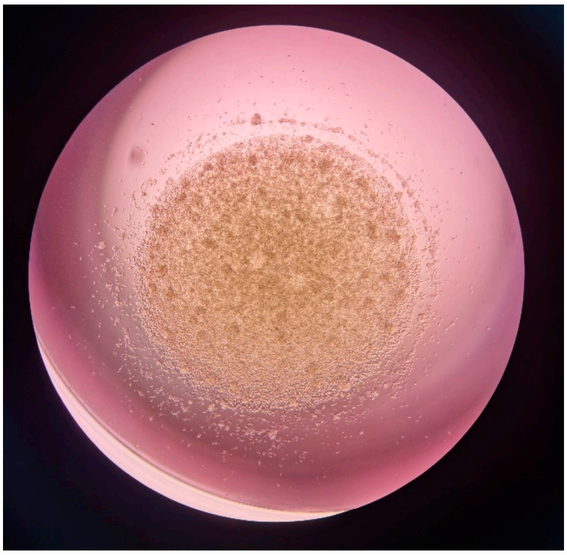

Plate 4

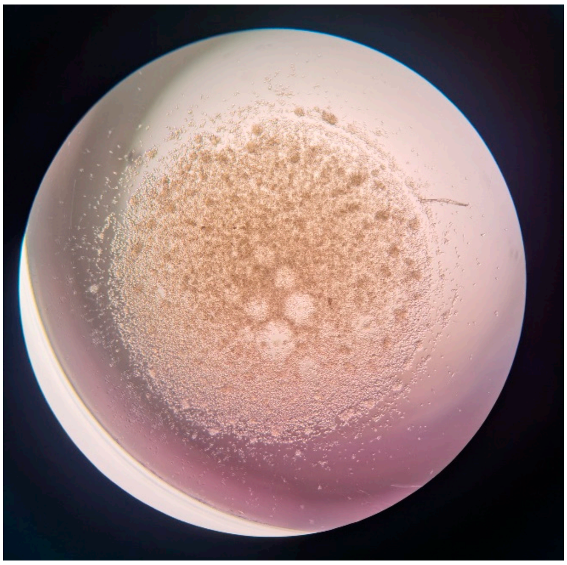

Figure S1J. **BV2 with HA35**

Plate 1

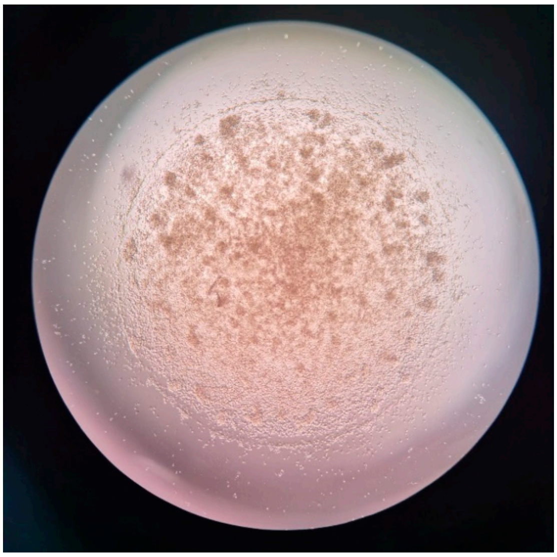

Plate 2

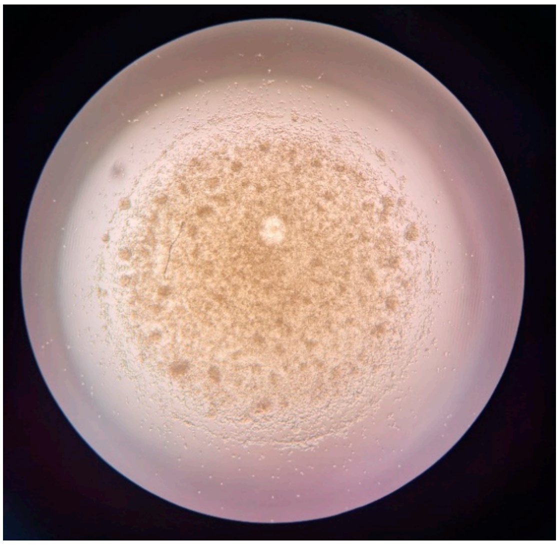

Plate 3

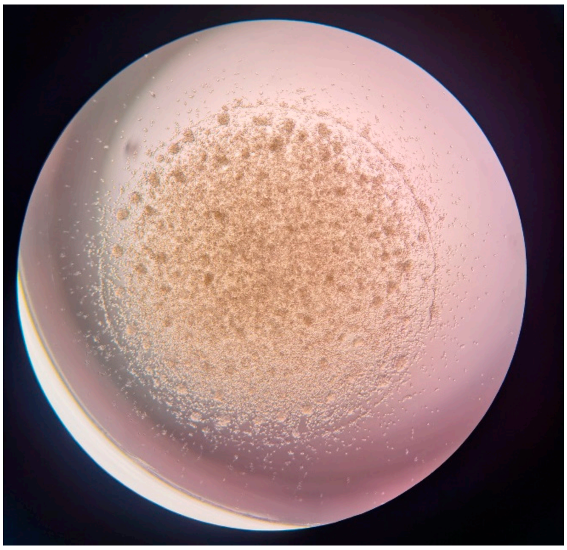

Plate 4

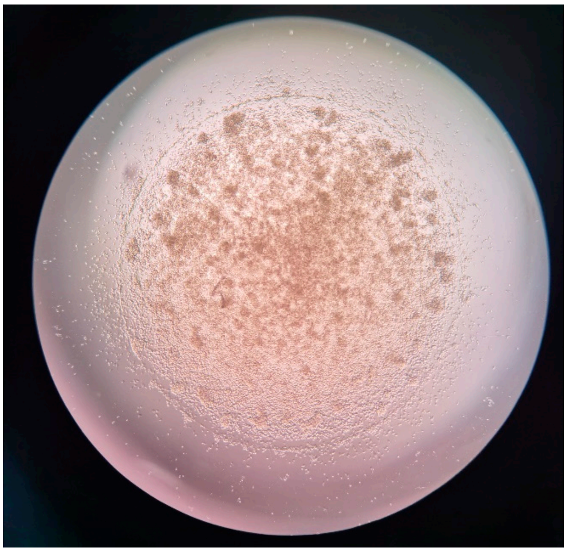

Figure S1K. **BV2 with HA1600**

Plate 1

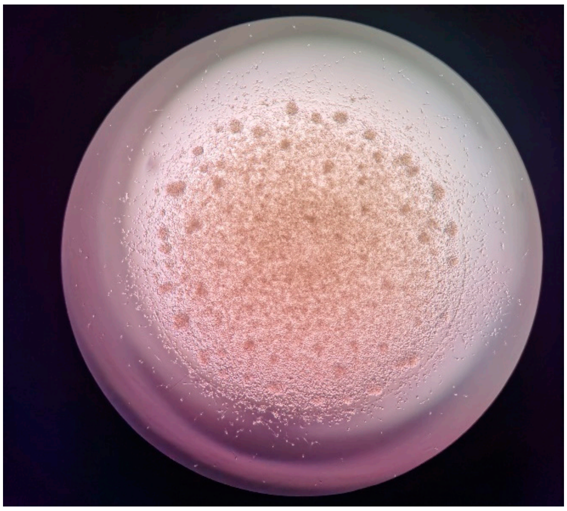

Plate 2

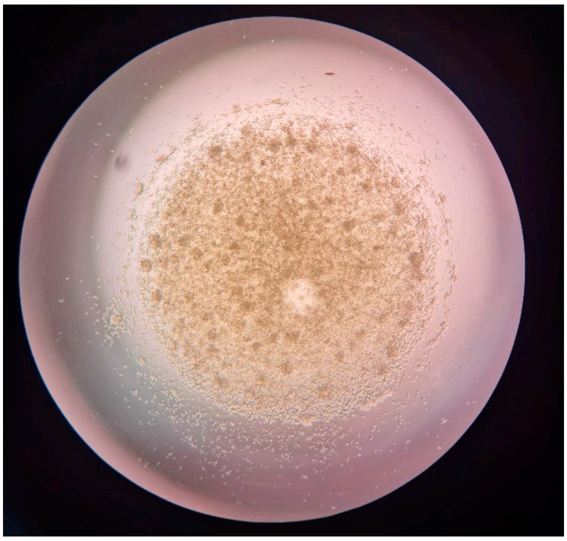

Plate 3

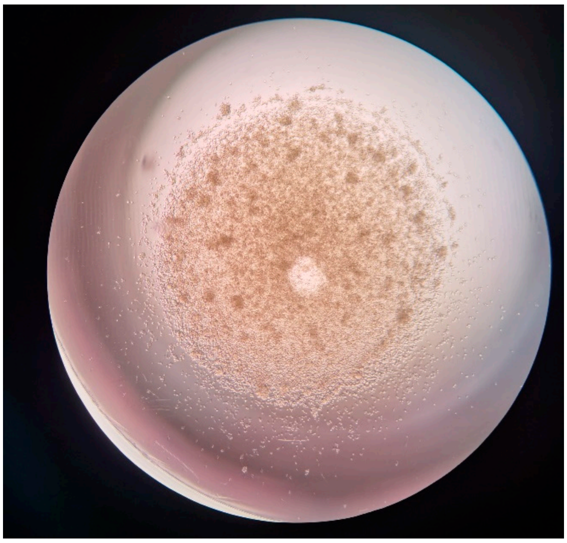

Plate 4

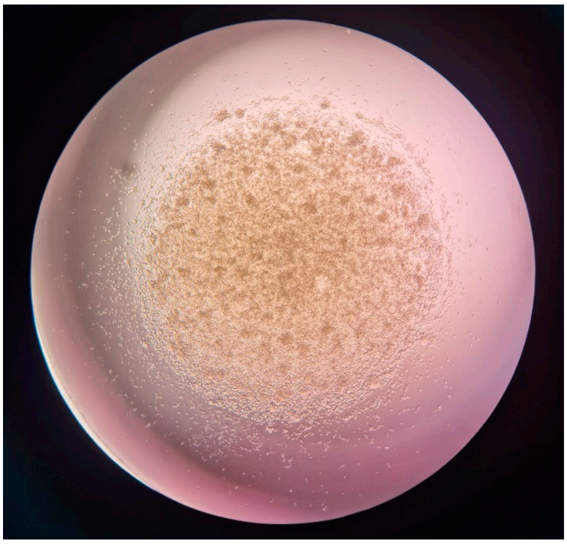

Figure S1L. **BAW264.7 Blank Control**

Plate 1

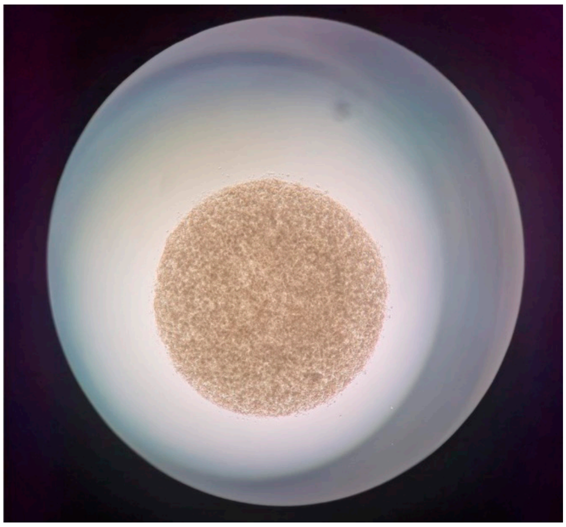

Plate 3

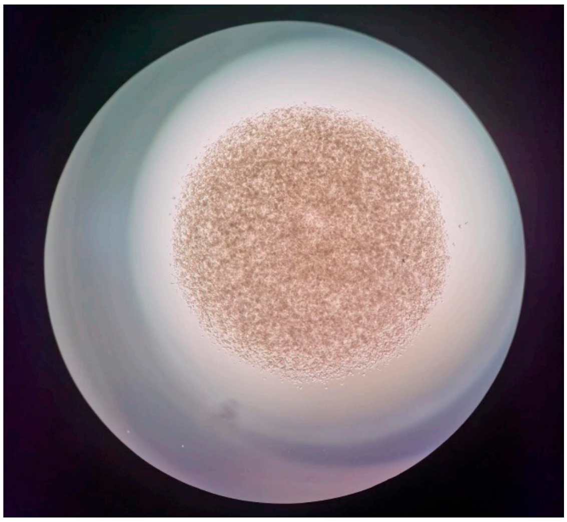

Plate 2

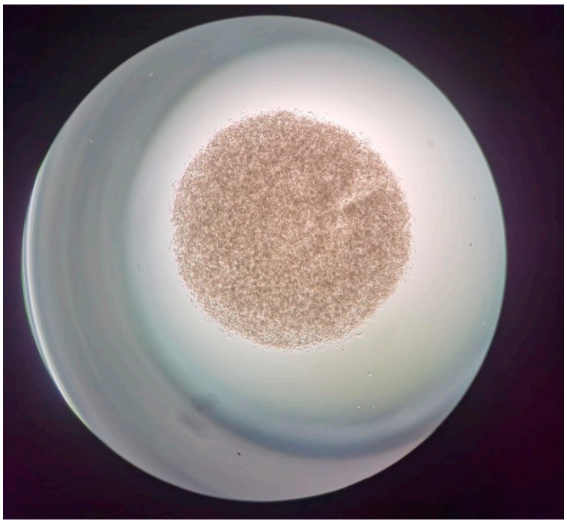

Plate 4

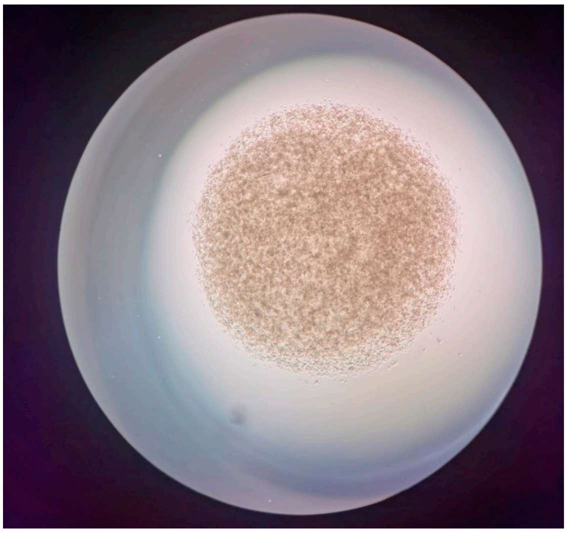

Figure S1M. **BAW264.7 with CD44**

Plate 1

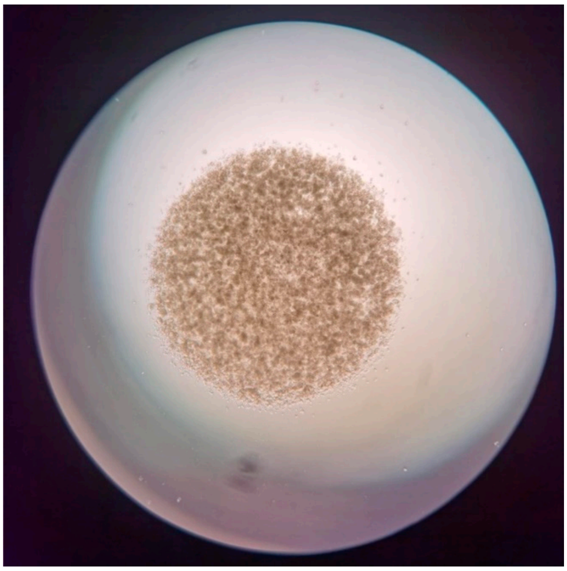

Plate 3

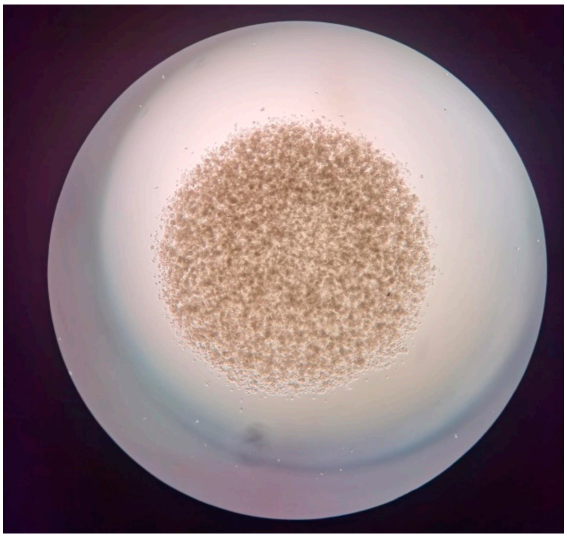

Plate 2

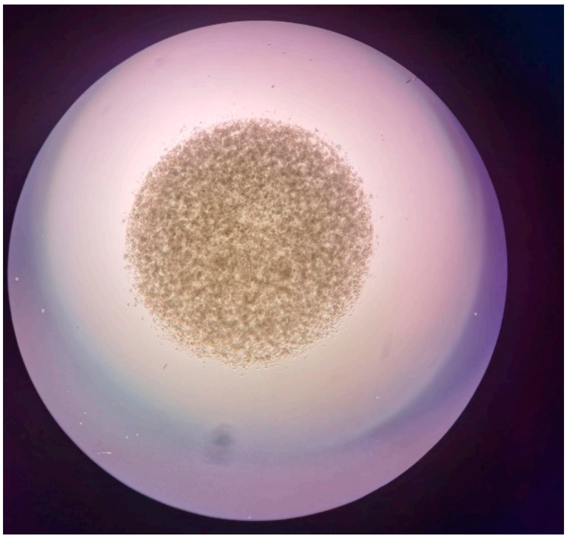

Plate 4

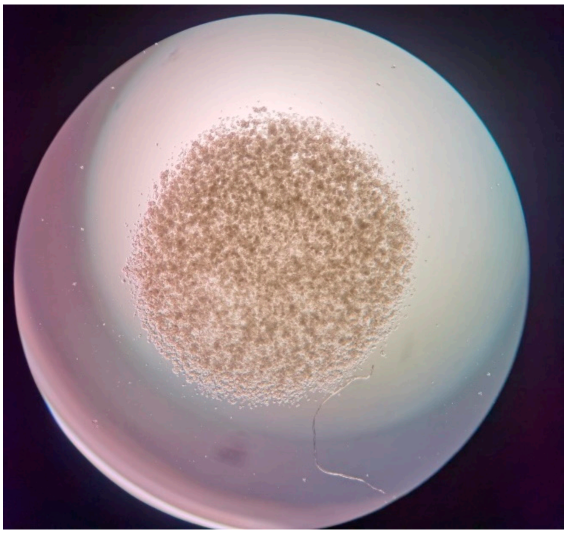

Figure S1N. **BAW264.7 fMLP**

Plate 1

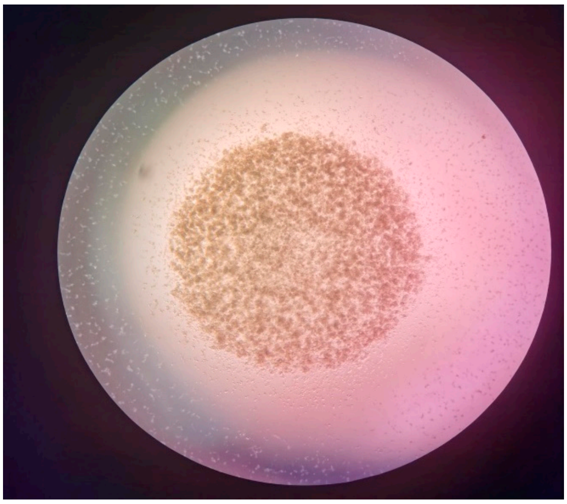

Plate 2

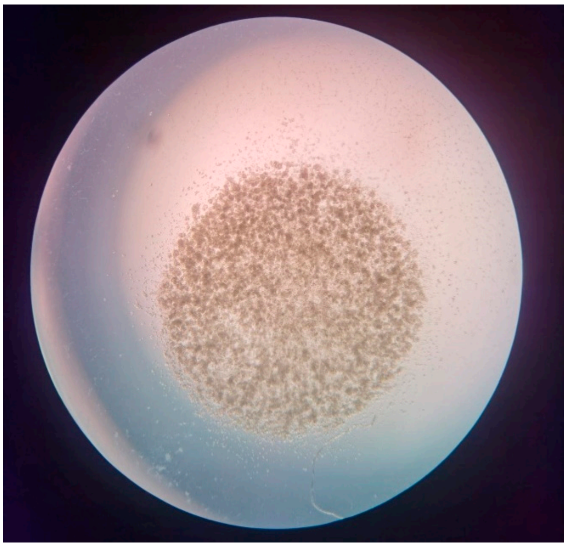

Plate 3

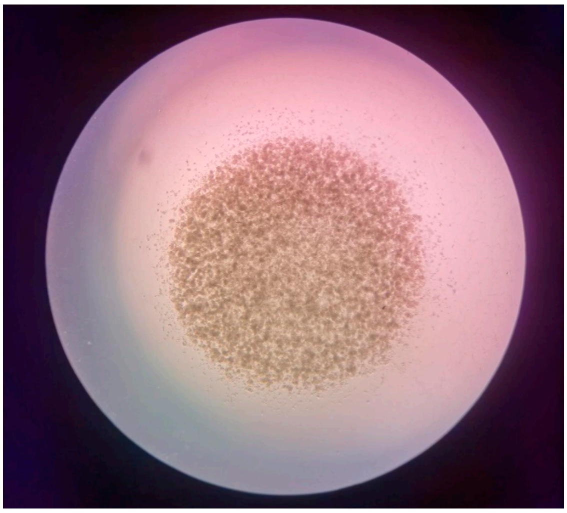

Plate 4

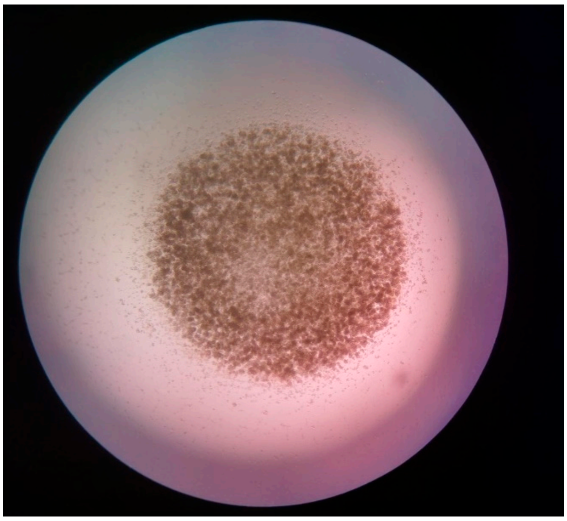

Figure S1O. **BAW264.7 with HA35**

Plate 1

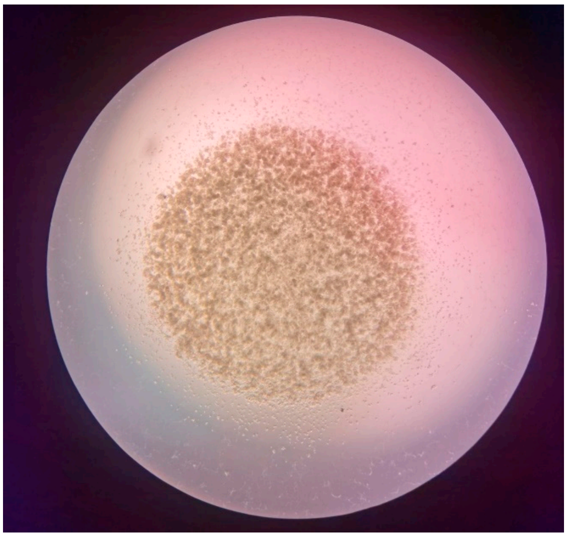

Plate 2

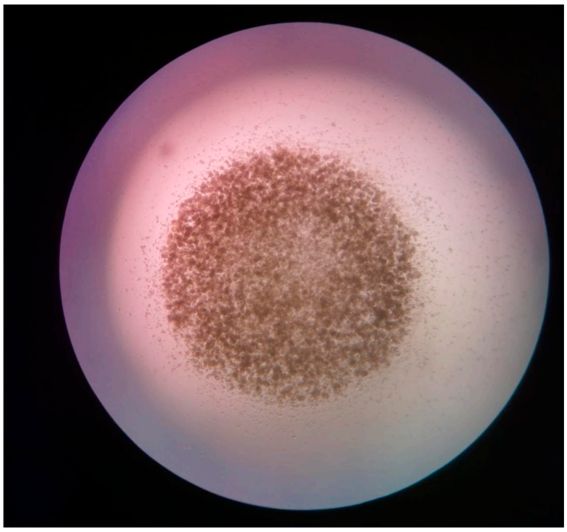

Plate 3

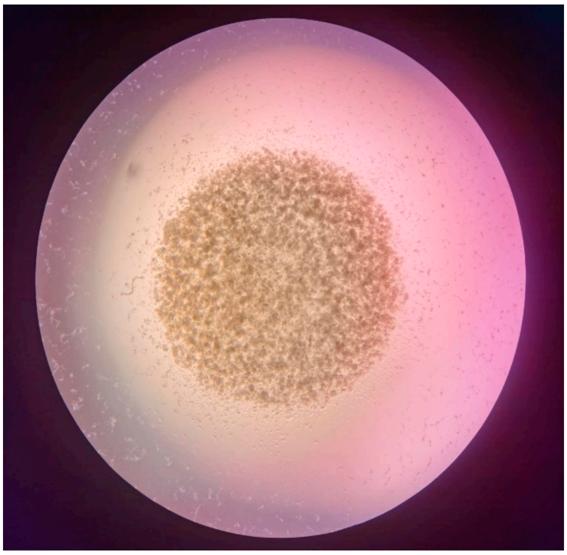

Plate 4

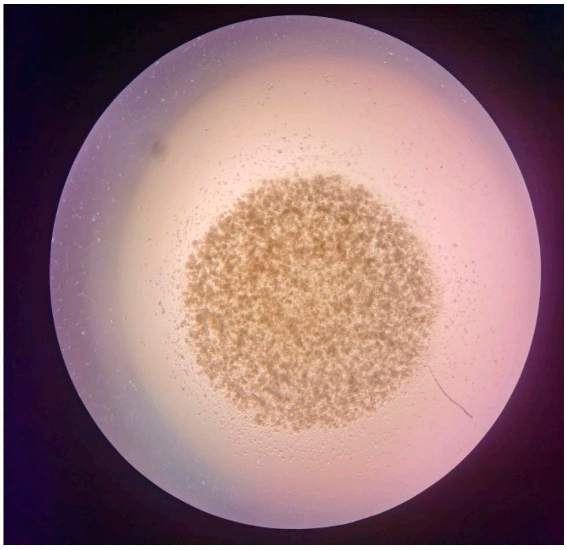

Figure S1P. **BAW264.7 with HA35 + anti-CD44**

Plate 1

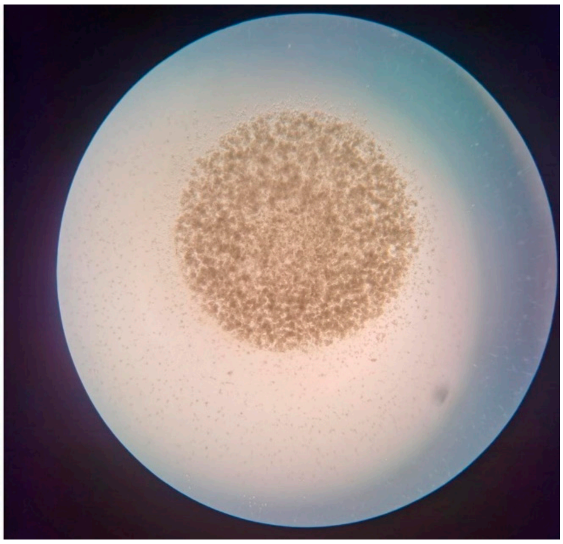

Plate 3

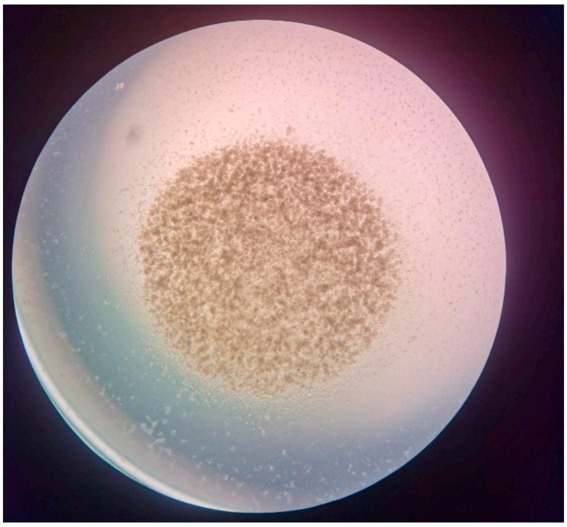

Plate 2

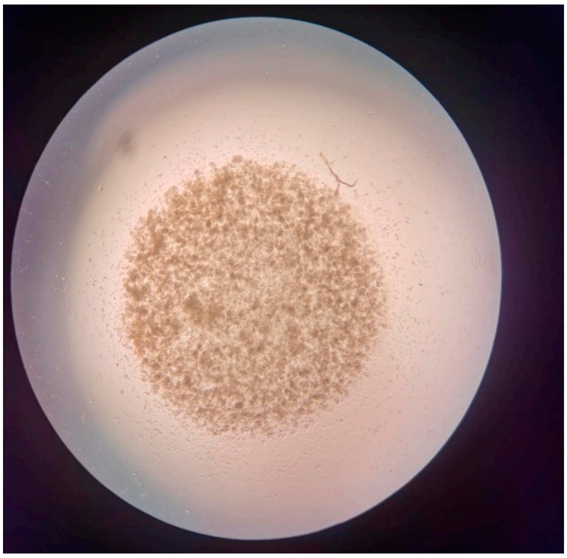

Plate 4

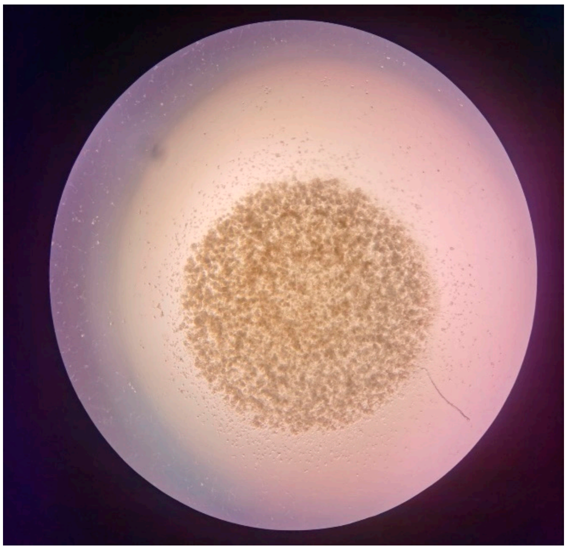

Figure S1Q. **BAW264.7 HA1600**

Plate 1

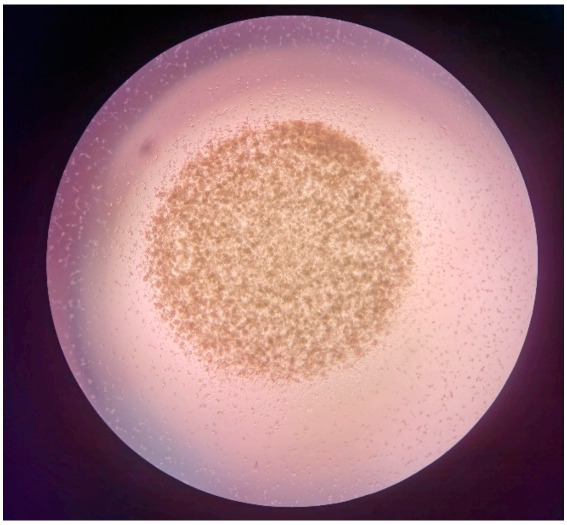

Plate 3

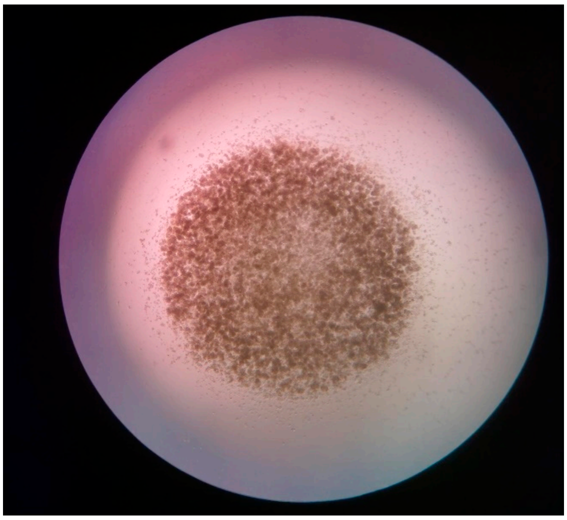

Plate 2

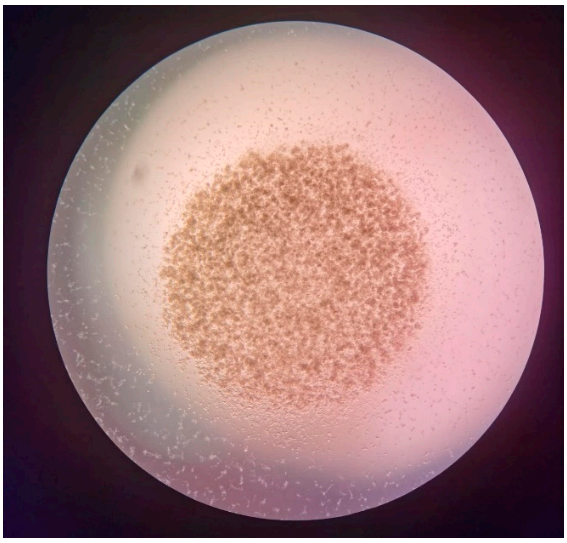

Plate 4

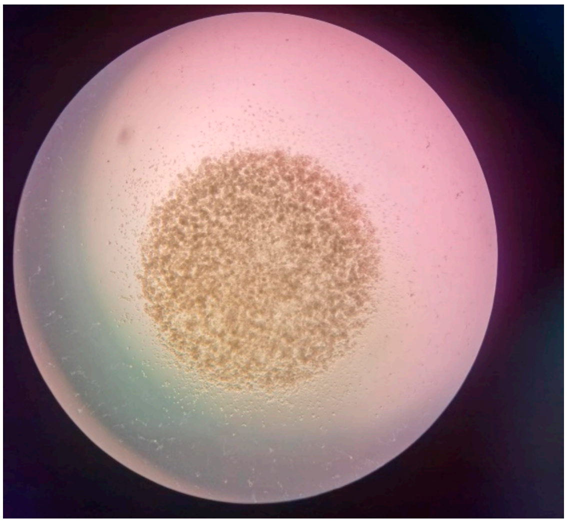

Figure S1R. **BAW264.7 HA1600 + anti-CD44**

Plate 1

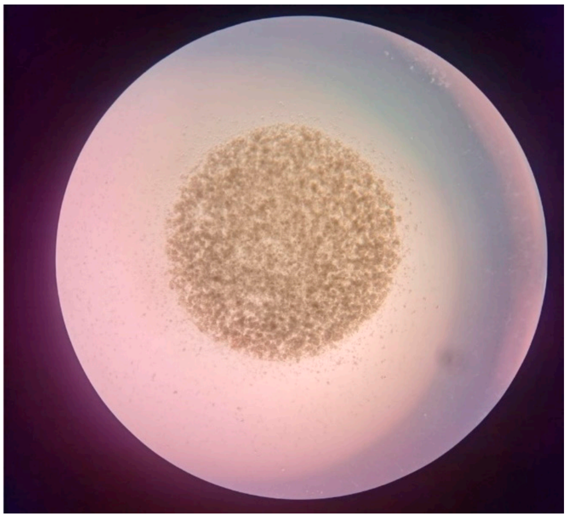

Plate 3

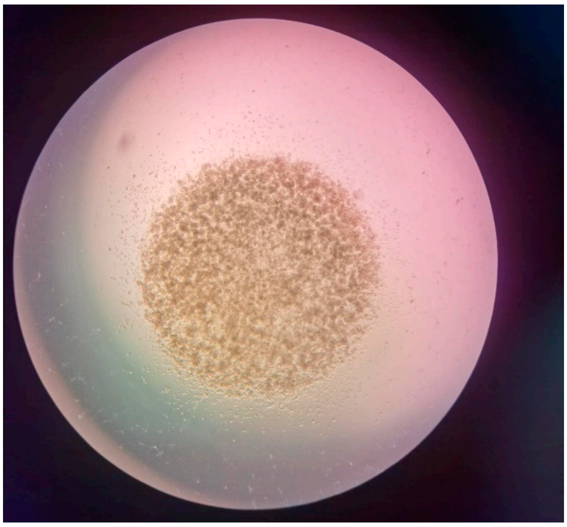

Plate 2

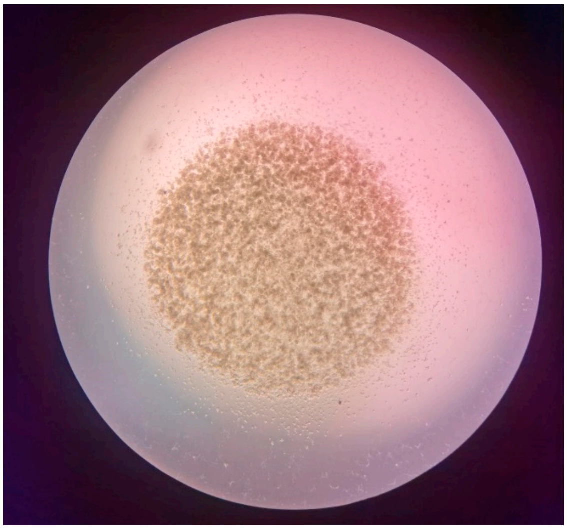

Plate 4

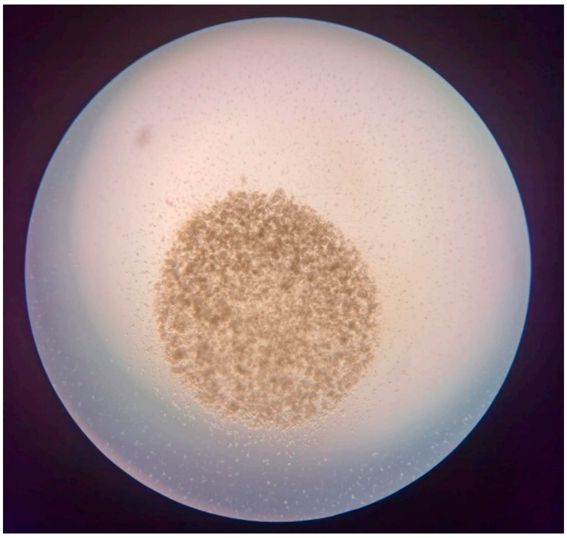

Figure S1S. **T9 cell Blank Control**

Plate 1

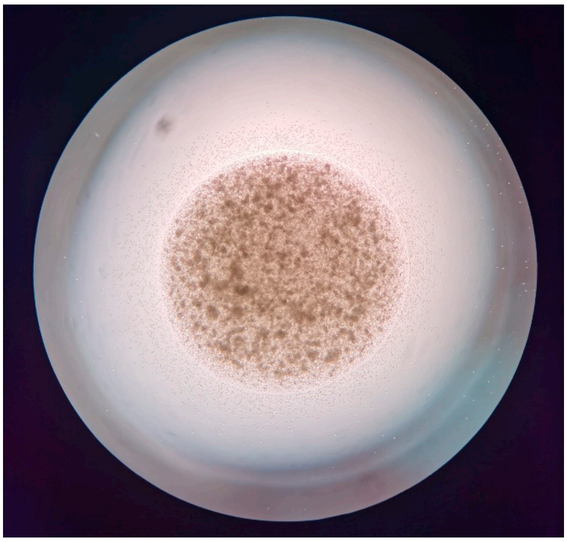

Plate 3

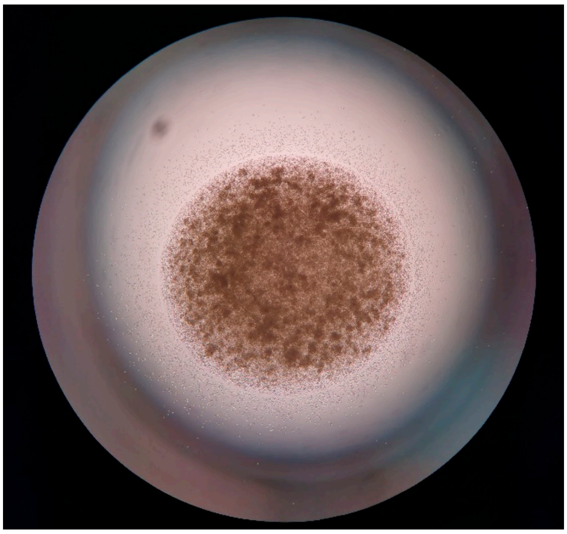

Plate 2

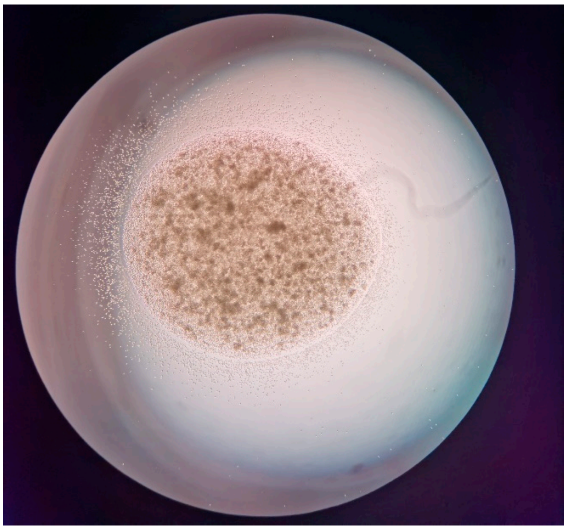

Plate 4

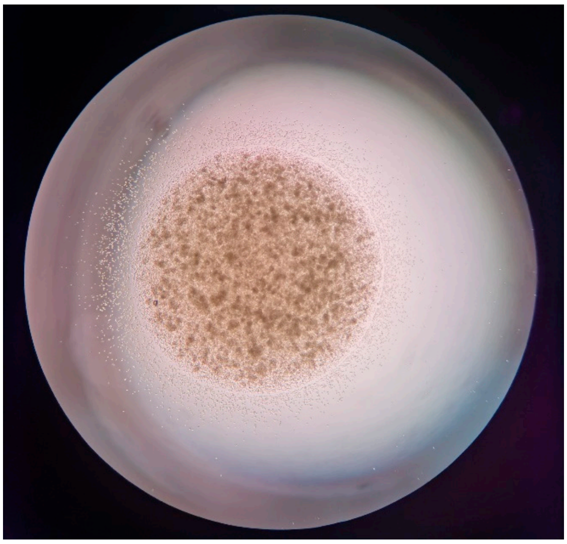

Figure S1T. **T9 anti-CD44**

Plate 1

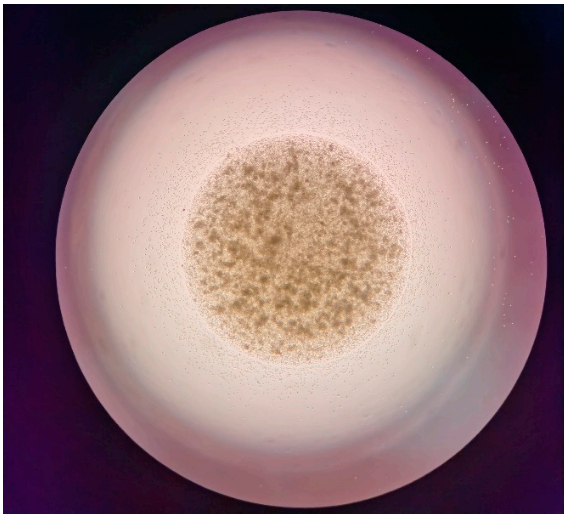

Plate 3

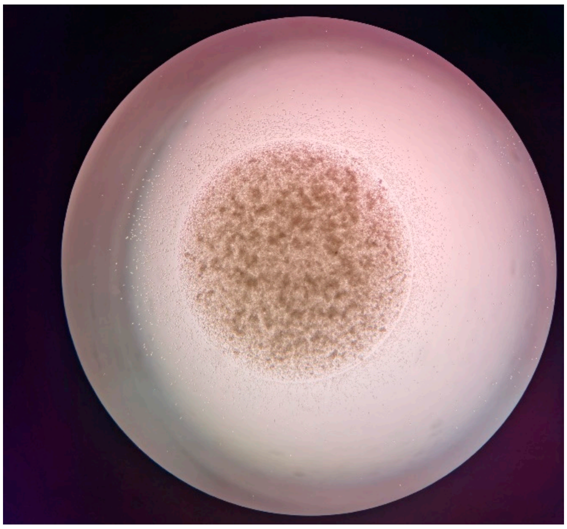

Plate 2

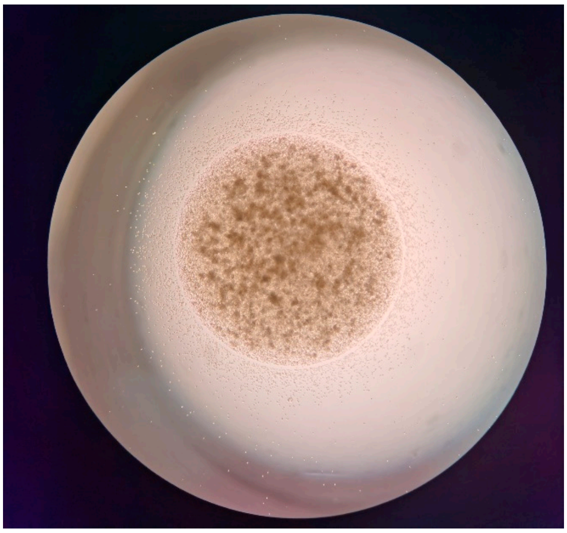

Plate 4

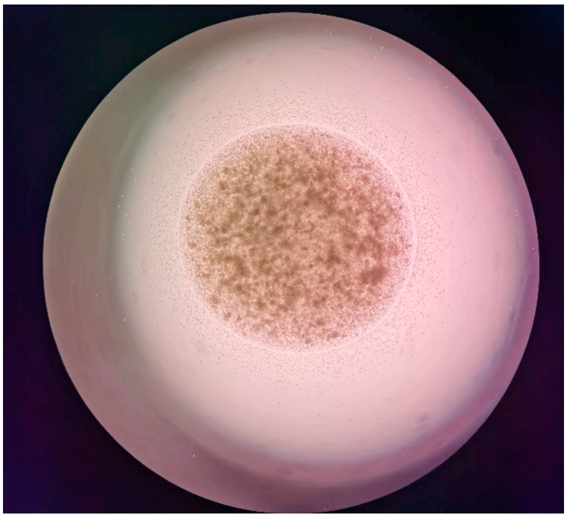

Figure S1U. **T9 with fMLP**

Plate 1

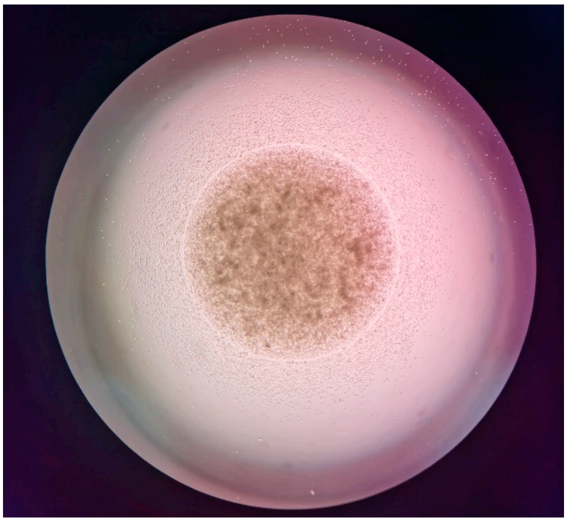

Plate 2

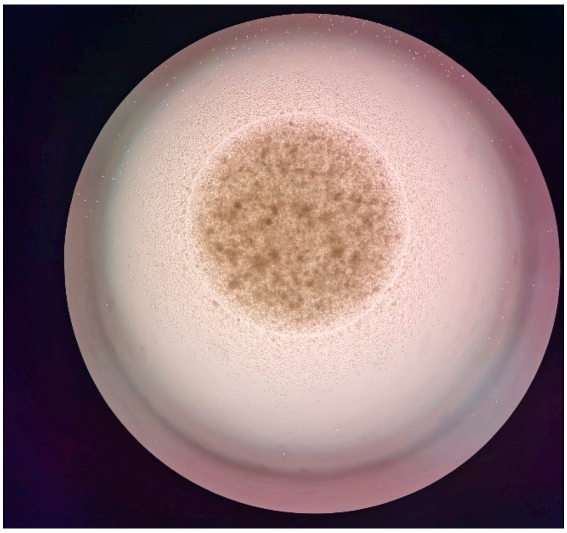

Plate 3

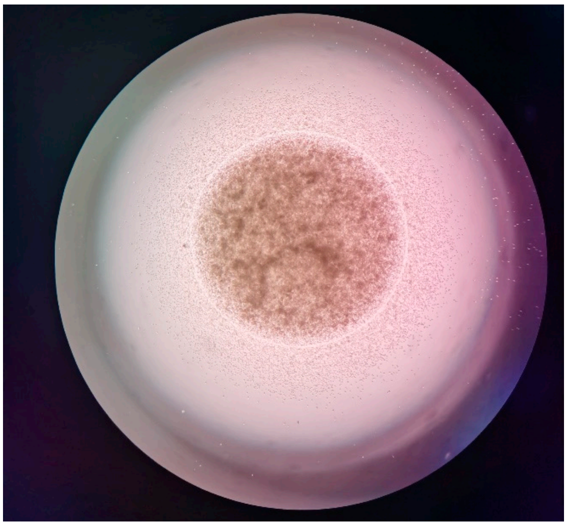

Plate 4

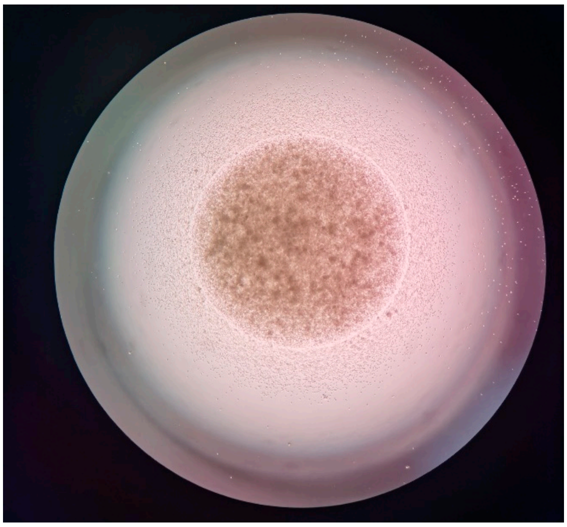

Figure S1V. **T9 with HA35**

Plate 1

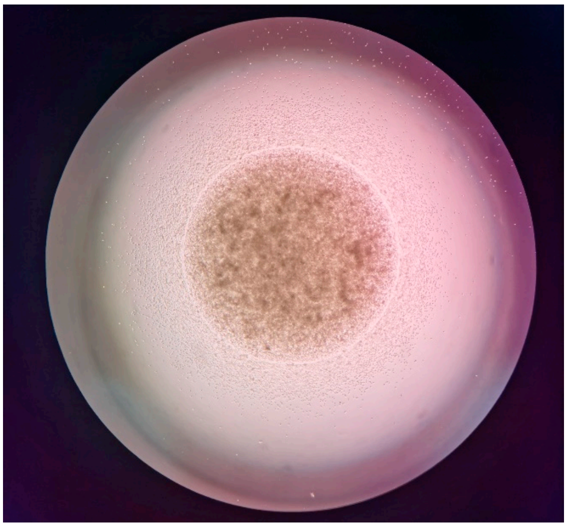

Plate 3

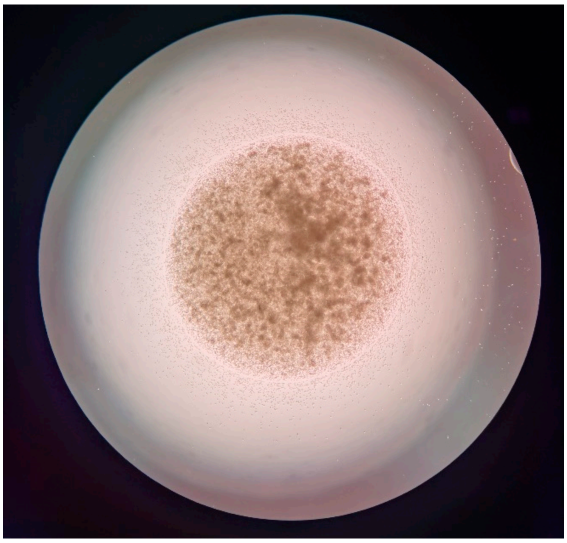

Plate 2

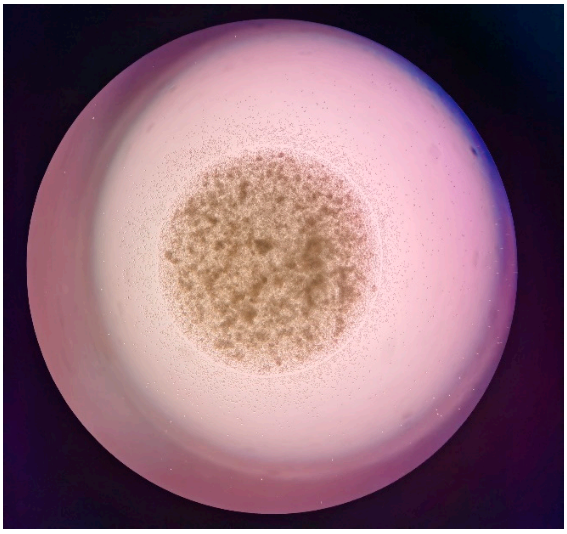

Plate 4

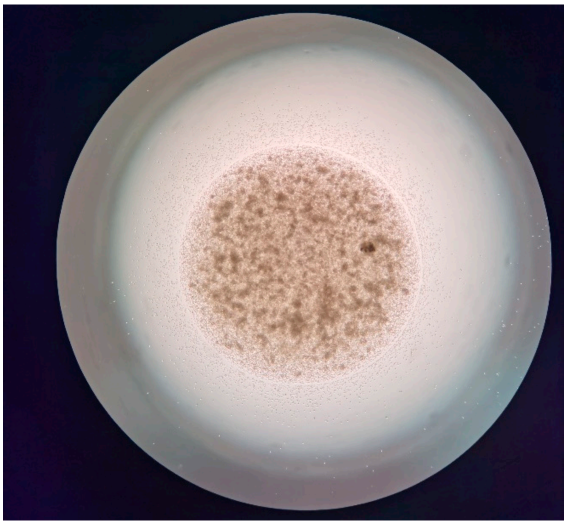

Figure S1W. **T9 with HA35 + anti-CD44**

Plate 1

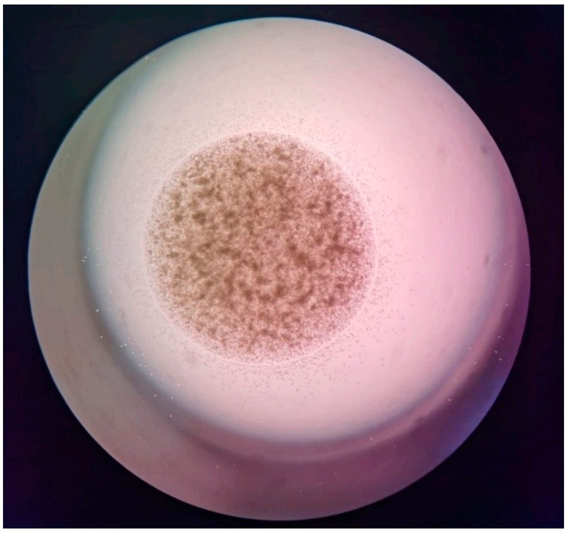

Plate 3

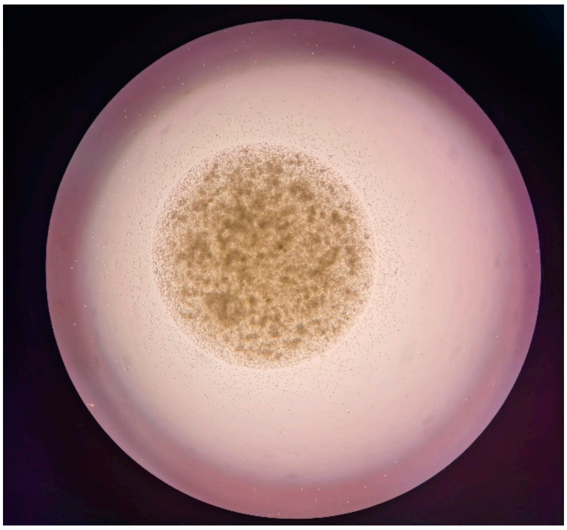

Plate 2

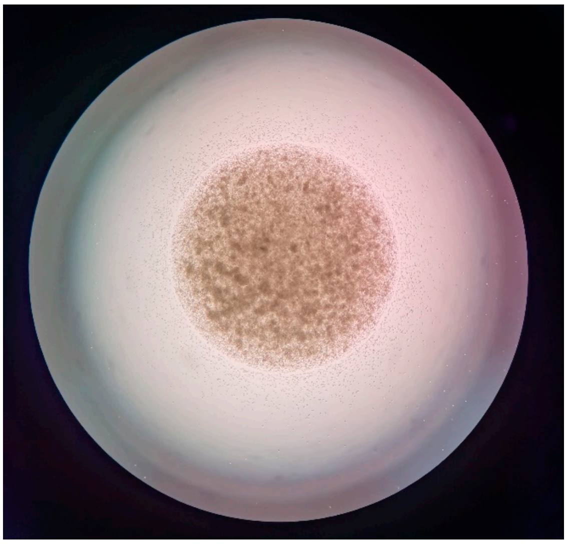

Plate 4

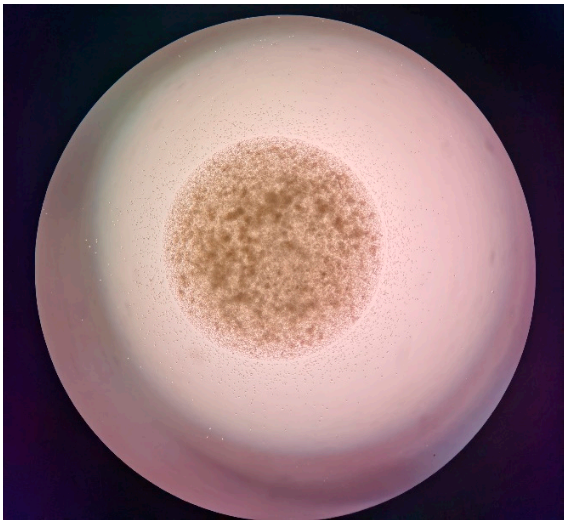

Figure S1X. **T9 with HA1600**

Plate 1

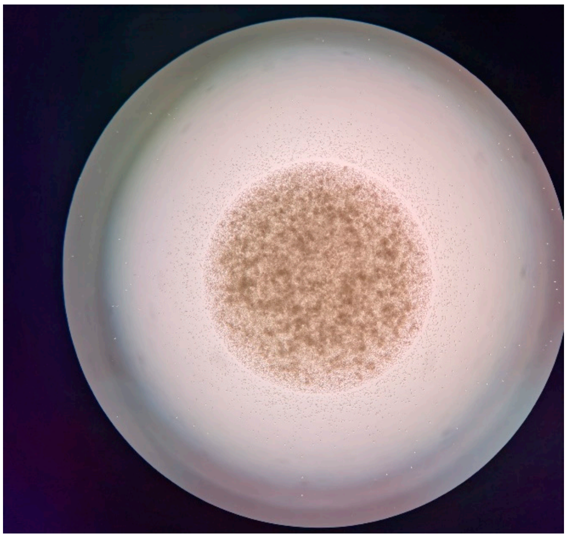

Plate 3

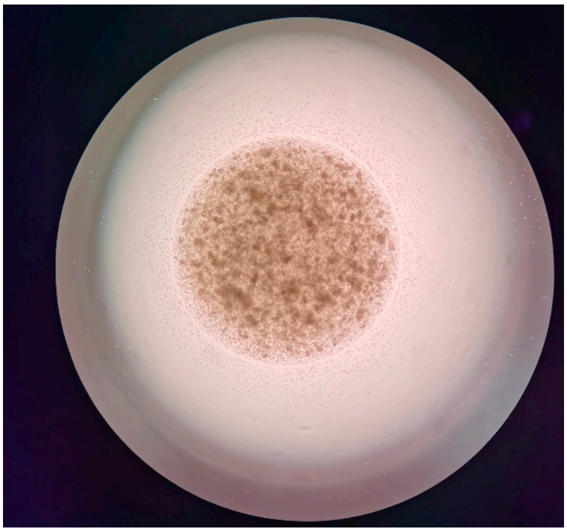

Plate 2

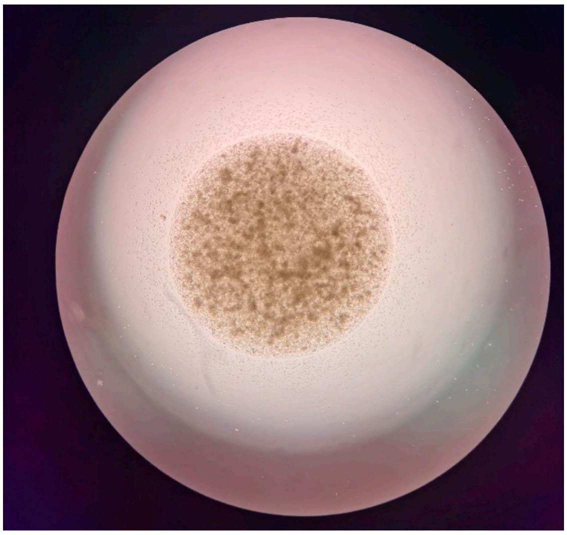

Plate 4

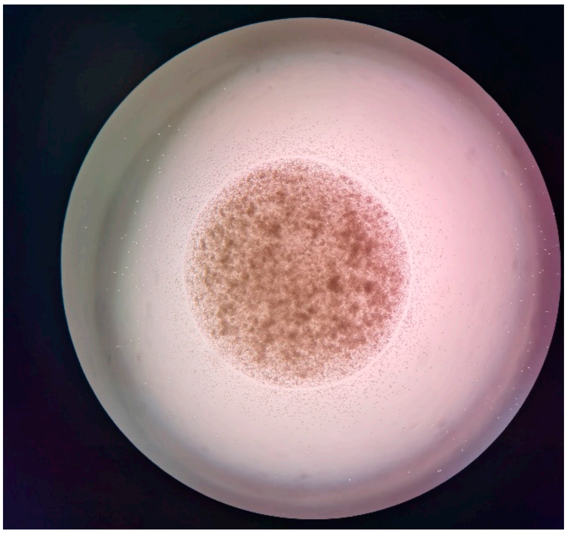

Figure S1Y. **T9 with HA1600 + anti-CD44**

Plate 1

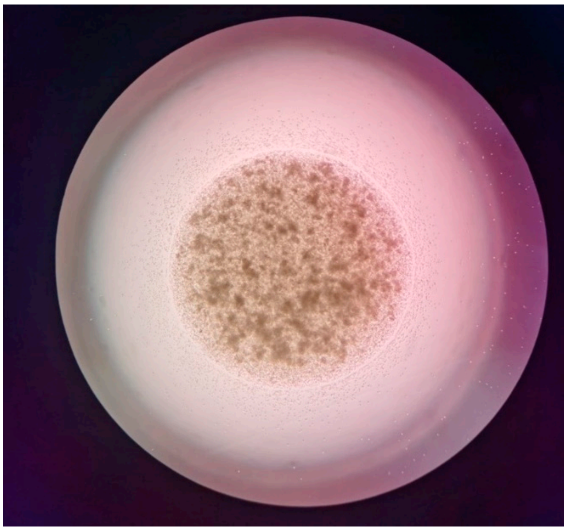

Plate 3

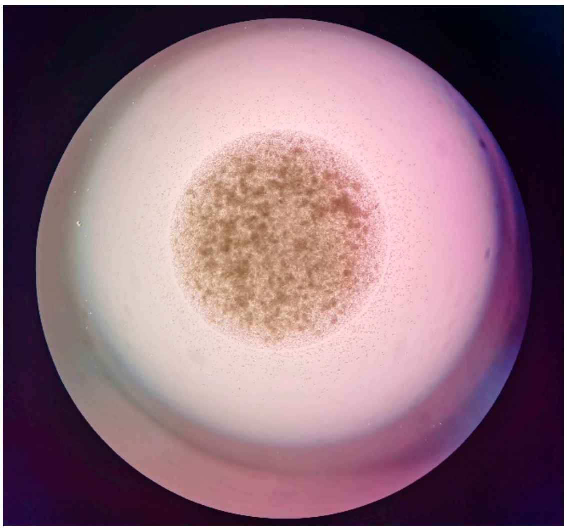

Plate 2

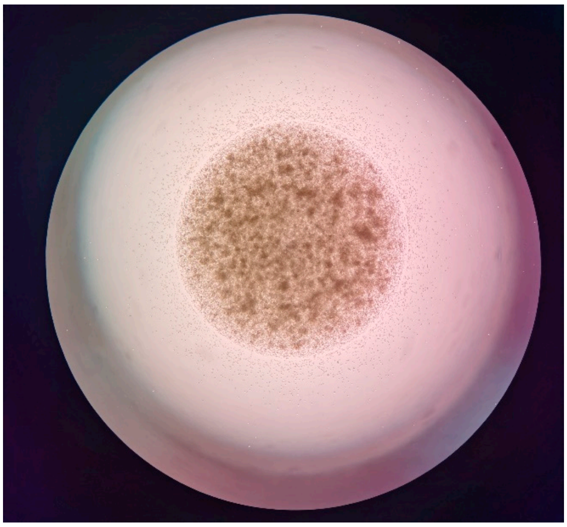

Plate 4

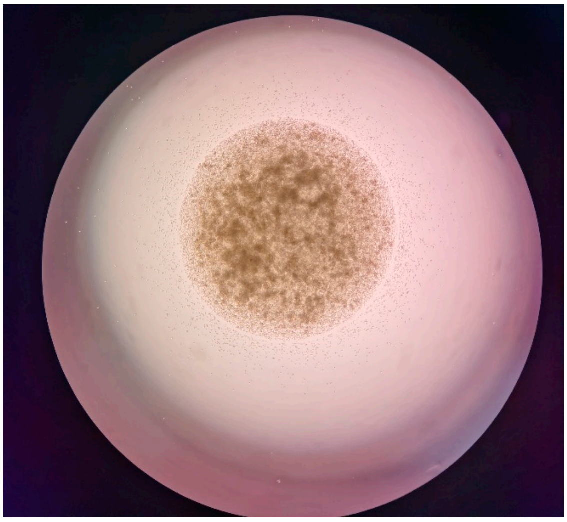

Supplementary Data II

Figure S2. ^{125}I-HA35 lymphatic imaging in healthy C57B/6J mice

Mice 2-4 received a 20-25 µCi ^{125}I-HA35 injection into the hind paws. Mouse 5 was intradermally injected with 25 µCi ^{125}I-HA35 into all four paws. Whole-body iQID images of the four mice were collected with 5-minute acquisitions at 180 minutes post-injection. Each mouse was anesthetized with 1-2% isoflurane and placed directly on the iQID camera for imaging acquisition. At the end of the imaging session, the mouse was euthanized, and tissue biodistribution measurements were taken to determine the percent injected dose per gram of tissue (%ID/g). **Figure S2** shows the iQID images from Mouse 2-5.

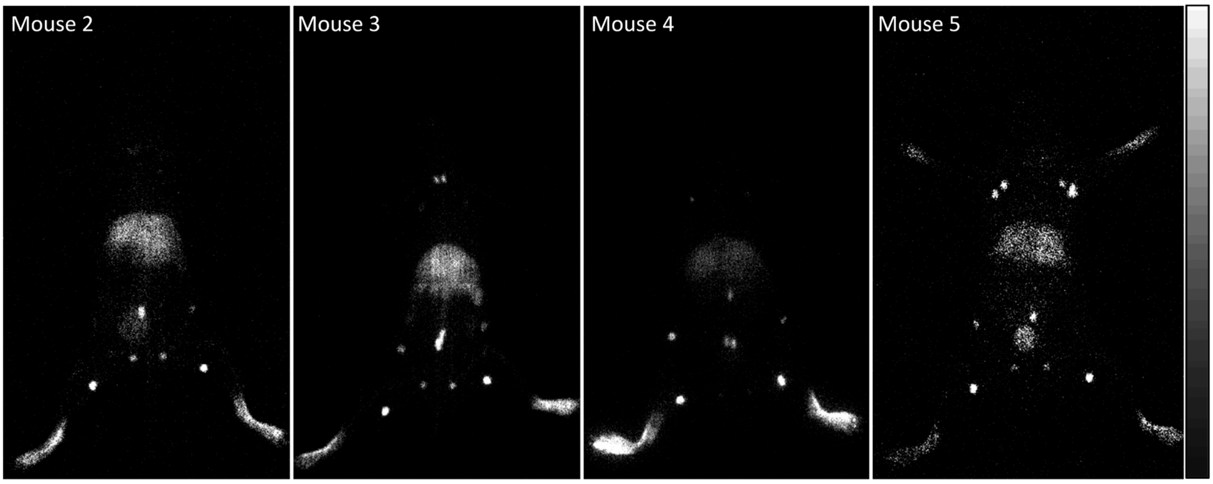

Figure S2. iQID images of ^{125}I-HA35 collected at 180 minutes post intradermal injections in the hind paws of C57BL/6J mice 2-4 and in all four paws of Mouse 5. The planar iQID image was acquired for each mouse over 5 minutes. Following the intradermal injection of ^{125}I-HA35 into the hind paws, the popliteal, inguinal, lumbar, iliac, sacral, pancreaticoduodenal, and cervical lymph nodes were primarily visualized, displaying varied levels of radioactivity. Axillary, brachial, and mandibular lymph nodes also became visible following the four-paw injection, as compared to the hind-paw injection only. The radioactive intensity in the major lymph nodes was higher than that in the spleen and liver. Low or moderate radioactivity was observed in the bladders.

Supplementary Data III

Figure S3. Imaging of ^{99m}Tc-HA35 Biodistribution in healthy C57B/6J mice

^{99m}Tc-HA35 (0.8-1.2 mCi) was intravenously injected into five C57BL/6J mice (aged 6-8 weeks). Under anesthesia with 1-2% isoflurane, whole-body images of the mice were obtained using the iQID camera with a 15-minute acquisition time. Mouse 1 was imaged at 30 minutes, 3 hours, and 21 hours after the injection of ^{99m}Tc-HA35 to assess whether there were any biodistribution changes related to radioactivity released from the ^{99m}Tc-labeled HA backbone, potentially due to the instability of ^{99m}Tc-HA35. Mice 2-5 were imaged only at 3 hours post-injection. Images of Mouse 1 acquired at 30 minutes and 21 hours are presented in **Figure 7** of the manuscript. **Supplementary Figure 1** shows the consistent biodistribution of ^{99m}Tc-HA35 in all five mice at 3 hours post intravenous administration. ^{99m}Tc-HA35 was predominantly localized in the liver, and a detectable amount of radioactivity was observed in the bladders. No significant radioactive uptake was observed in the salivary glands, stomach, or thyroid, which is usually indicative of free ^{99m}Tc pertechnetate released from labeled molecules. The iQID imaging results indicate that the radioactive accumulation in the liver was a consequence of the exogenously administered stable HA.

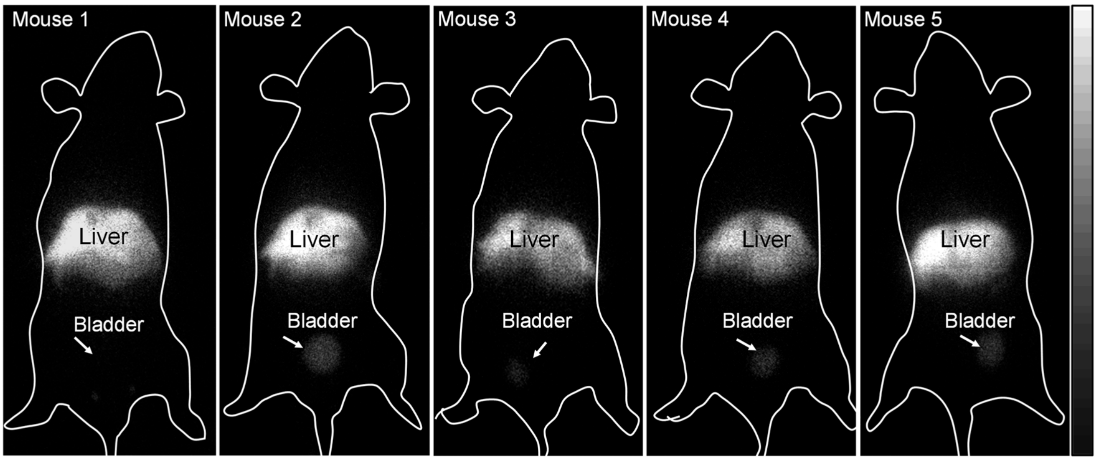

Figure S3: iQID images of ^{99m}Tc-HA35 in five C57BL/6J mice, acquired 3 hours post-injection. The images show predominant localization of radioactivity in the liver. The bladder (arrow) is also detectable with low levels of radioactivity.
